# The effect of developmental variation on expression QTLs in a multi parental *Caenorhabditis elegans* population

**DOI:** 10.1093/g3journal/jkad273

**Published:** 2023-11-28

**Authors:** Abraham L van Eijnatten, Mark G Sterken, Jan E Kammenga, Harm Nijveen, Basten L Snoek

**Affiliations:** Theoretical Biology and Bioinformatics, Utrecht University, Padualaan 8,3584 CH Utrecht, The Netherlands; Laboratory of Nematology, Wageningen University, Droevendaalsesteeg 1, 6708 PB Wageningen, The Netherlands; Laboratory of Nematology, Wageningen University, Droevendaalsesteeg 1, 6708 PB Wageningen, The Netherlands; Laboratory of Bioinformatics, Wageningen University, Droevendaalsesteeg 1, Radix West, Building 107, 6708 PB Wageningen, The Netherlands; Theoretical Biology and Bioinformatics, Utrecht University, Padualaan 8,3584 CH Utrecht, The Netherlands

**Keywords:** *C. elegans*, genetics, eQTL mapping, developmental age

## Abstract

Regulation of gene expression plays a crucial role in developmental processes and adaptation to changing environments. expression quantitative trait locus (eQTL) mapping is a technique used to study the genetic regulation of gene expression using the transcriptomes of recombinant inbred lines (RILs). Typically, the age of the inbred lines at the time of RNA sampling is carefully controlled. This is necessary because the developmental process causes changes in gene expression, complicating the interpretation of eQTL mapping experiments. However, due to genetics and variation in ambient micro-environments, organisms can differ in their “developmental age,” even if they are of the same chronological age. As a result, eQTL patterns are affected by developmental variation in gene expression. The model organism *Caenorhabditis elegans* is particularly suited for studying the effect of developmental variation on eQTL mapping patterns. In a span of days, *C. elegans* transitions from embryo through 4 larval stages to adult while undergoing massive changes to its transcriptome. Here, we use *C. elegans* to investigate the effect of developmental age variation on eQTL patterns and present a normalization procedure. We used dynamical eQTL mapping, which includes the developmental age as a cofactor, to separate the variation in development from genotypic variation and explain variation in gene expression levels. We compare classical single marker eQTL mapping and dynamical eQTL mapping using RNA-seq data of ∼200 multi-parental RILs of *C. elegans*. The results show that (1) many eQTLs are caused by developmental variation, (2) most trans-bands are developmental QTLs, and (3) dynamical eQTL mapping detects additional eQTLs not found with classical eQTL mapping. We recommend that correction for variation in developmental age should be strongly considered in eQTL mapping studies given the large impact of processes like development on the transcriptome.

## Teaser

van Eijnatten *et al*. use a multi-parental RIL population of the model organism *C. elegans* to explore the impact of developmental age variation on eQTL mapping patterns. By employing dynamical eQTL mapping, they separate developmental age variation from genotypic variation to explain variation in gene expression levels. Their results show that many eQTLs are caused by developmental variation, that most trans-bands are associated with developmental age, and that dynamical eQTL mapping detects additional eQTLs not found with classical eQTL mapping.

## Introduction

Regulation of gene expression is a key mechanism underlying the huge diversity of phenotypes, adaptations, and developmental stages within and across species. Understanding gene expression regulation provides insights into how organisms develop and adapt to changing environments (Reinke *et al*. 2000; Spencer *et al.* 2011; Araya *et al.* 2014; Francesconi and Lehner 2014; Snoek *et al.* 2014; Boeck *et al.* 2016; Jovic *et al.* 2017). The genetic regulation of gene expression can be investigated through expression quantitative trait locus (eQTL) mapping, also called genetical genomics (Jansen and Nap 2001). In this statistical procedure, polymorphic regions (eQTLs) are associated with variation in gene expression, pinpointing potential regulatory regions. The nematode *Caenorhabditis elegans* is often used in eQTL studies and has a small, well-annotated genome in addition to well-documented genetic variation (Snoek *et al.* 2013, 2020; Sterken *et al.* 2015; Evans *et al.* 2021; Andersen and Rockman 2022). Its tolerance of cryopreservation, large brood size, and self-fertilizing ability allow for the construction of perpetual recombinant inbred lines (RILs) derived from genetically diverse *C. elegans* isolates (Gaertner and Phillips 2010; Sterken *et al.* 2015; Evans *et al.* 2021). RILs are homozygous for almost all loci and constitute a genetic mosaic of the parental genotypes, thereby increasing the resolution and power of methods for eQTL detection (Andersen and Rockman 2022). The above qualities make *C. elegans* an exceptional model system for genetical genomics.

The developmental process in *C. elegans* is associated with massive and rapid changes in gene expression (Snoek *et al.* 2014; Hashimshony *et al.* 2015; Levin *et al.* 2016). Many genes are expressed in a developmental stage-specific manner (Reinke *et al.* 2000; McCarroll *et al.* 2004; Viñuela *et al.* 2010, 2012; Boeck *et al.* 2016). Others are up- and downregulated cyclically as *C. elegans* transitions through the 4 larval stages (Kim *et al.* 2013; Hendriks *et al.* 2014; Meeuse *et al.* 2020). The speed of development, as well as the process of aging, varies between individual *C. elegans* strains due to stochastic and micro-environmental factors, maternal age, and variation in the genetic background (Viñuela *et al.* 2010, 2012; Volkers *et al.* 2013; Francesconi and Lehner 2014; Snoek *et al.* 2014, 2019; Perez *et al.* 2017; Bulteau and Francesconi 2022). Due to the impact of the developmental process on gene expression combined with the interaction with the genetic background, eQTL patterns can be affected by uncontrolled developmental variation (Viñuela *et al.* 2010; Francesconi and Lehner 2014; Snoek *et al.* 2014). To conceptualize developmental variation, it is useful to distinguish between the chronological age and the developmental age. The chronological age is simply the measured age of the organism, whereas the developmental age represents how far the organism has progressed along the developmental process. Because variation in developmental speed and thus developmental age depends partly on genetic factors, it is intrinsic to populations used for genetic mapping. Careful synchronization of worms at a particular developmental checkpoint can help to reduce but does not eliminate developmental age variation (Ben-David *et al.* 2021). As a result, the eQTL mapping procedure could attribute expression variation resulting from variation in developmental age to a genetic polymorphism. Conversely, developmental age variation could obscure the effects of the genetic background on gene expression.

One way to deal with the confounding effect of the developmental process in eQTL mapping is to apply a normalization procedure (Viñuela *et al.* 2012; Jovic *et al.* 2019). Another approach is to make the effects of developmental variation on gene expression explicit by including the developmental age as a cofactor in the statistical model for eQTL mapping (dynamical eQTL mapping; Francesconi and Lehner 2014). The latter approach is more informative because it explicitly models how eQTLs affect the dynamics of gene expression, rather than just the mean over the developmental ages of the inbred lines. A seminal paper by Francesconi and Lehner (Francesconi and Lehner 2014) showed that dynamical eQTL mapping using the quantified developmental age can be leveraged to detect additional eQTLs. By relating the magnitude of gene expression to the developmental age, the authors showed that eQTLs can affect the expression dynamics over the course of the developmental process.

In this study, we aimed to quantify the difference between the classical eQTL mapping approach, which does not explicitly consider developmental variation, and dynamical eQTL mapping. To this end, we used RNA-seq data of ∼200 *C. elegans* multi-parental RILs (mpRILs) (Snoek *et al.* 2019, 2021). RNA-seq samples were obtained 48 h after synchronization by egg bleaching, ensuring there is no variation in chronological age within the population. At the time of RNA sampling, the mpRILs were in the L4 larval stage. We started by quantifying the developmental age directly from the gene expression data with a straightforward approach involving principal component analysis (PCA). Next, we performed eQTL mapping using linear models, both with and without the developmental age included as a cofactor. We compared these models qualitatively, by the distribution of eQTLs over the genome, and quantitatively, by the number of eQTLs detected and the variance in gene expression attributed to the eQTLs. We show that most, but not all, *trans*-bands (regulatory hotspots) result from a shared association with the developmental age between the SNP marker and transcript levels of genes involved in developmental processes. Moreover, we present evidence that such hotspot loci affect gene expression through their effect on developmental speed. We also show how comparing the results from models with and without the developmental age can help to distinguish between loci linked to the developmental age and other regulators of gene expression.

## Methods

All analyses were performed using R version 4.1.3 (R Core Team 2022). Plots were generated using the ggplot2 package (Wickham 2016). Pearson correlations were calculated using the cor() function from base R.

### Data and pre-processing

We used data from 199 mpRILs (Volkers *et al.* 2013; Snoek *et al.* 2019, 2021). The mpRILs were derived from an advanced cross between 4 parental lines which were collected from the French regions of Orsay (2 lines) and Santeuil (2 lines). The mpRILs were grown for 48 h after bleaching at 24°. We verified that our population was still in L4 by calculating the correlation of the transcriptome of the mpRILs with 2 L4 reference genomes and 2 young adult reference genomes from the N2 strain (L4, L4b, YA, and N2Yad-1 from the study by Boeck *et al.* 2016; Supplementary Fig. 14). The parental lines were excluded from the eQTL mapping procedure and all subsequent analysis unless explicitly mentioned. The data consist of fpkm values for 38,322 transcripts obtained using RNA-seq and a genetic map of 8,933 bi-allelic SNPs. Due to the SNPs being bi-allelic, the genotype at each marker was coded as 0 or 1 for homozygous individuals and 0.5 for heterozygous individuals. Before analysis, we filtered the gene expression data to retain the 12,029 transcripts with more than 20 non-zero fpkm values and a log_2_(mean fpkm) value higher than −5. Out of the 12,029 filtered transcripts, 2,341 are polycistronic, for a total of 15,224 genes in the filtered dataset. In all analyses we use the center log ratio (CLR) of gene expression, which for gene *j* and mpRIL *i* is calculated according to the following equation:


$$\mathrm{CL}R_{i,j}=\log_{2}\;\left(\frac{\left(\mathrm{fpk}m_{i,j}+1\right)}{\left(\frac{1}{n}\left(\sum_{i}^{n}\;\mathrm{fpk}m_{i,j}\right)+1\right)}\right)$$

### Developmental age

**Fig. 1.**
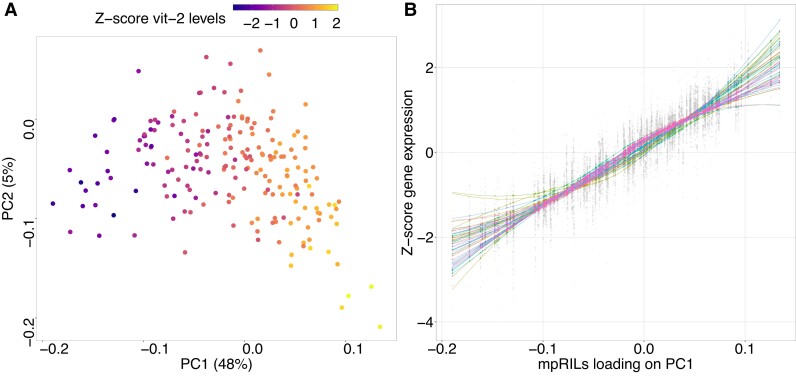
Developmental age estimation using PCA. a) PCA results. Points are the projections of the mpRILs on PC1 (*x*-axis) and PC2 (*y*-axis). Color indicates the *Z*-score of the CLR of fpkm values of *vit-2*, a developmental indicator gene (Snoek *et al*. 2014). b) Gene expression of the 53 developmental indicator genes from the study by Snoek *et al*. (2014) with expression in this dataset. The *x*-axis shows the projections of the mpRILs on PC1. The *y*-axis shows the *Z*-score of the CLR of fpkm values. Colored trendlines correspond to a generalized additive model fit to the expression values of each developmental indicator gene.

We quantified the developmental age by performing PCA on the filtered and normalized gene expression matrix [with the mpRILs as columns and the transcripts as rows, such that mpRILs have loadings on principal component (PC) 1 and transcripts have scores] using the prcomp() function from the stats package. The loadings of the mpRILs on PC1 were taken as a proxy for the developmental age in subsequent analysis. For Fig. 1, Supplementary Figs. 2–4 and 12, we calculated the *Z*-score of the CLR of the fpkm values by mean centering on 0 and scaling such that the expression of each gene has a standard deviation of 1 using the scale() function from base *R*.

We calculated developmental age estimates using the ae() function from the RAPToR package in *R*. We tried both the Cel_larval and Cel_larv_ya reference sets, obtaining a more even spread in distribution using the former. The ae() function allows for the possibility of specifying a prior but this did not provide a narrower distribution of the estimated ages, even when using a low standard deviation (1 h). We therefore estimated the ages of the mpRILs with the Cel_larval reference and no prior.

### eQTL mapping with linear models

We ran linear models using the FastLm() function from the RcppAramadillo package. We applied a single marker model (SMM, gene expression ∼ marker genotype), additive age model (AAM, gene expression ∼ marker genotype + PC1), and interaction model [IM, gene expression ∼ marker genotype (IMM term) + PC1 + marker genotype × PC1 (IMI term)] to perform pairwise tests between the filtered transcripts and all markers. *P*-Values were calculated for model terms using *t*-tests. For the IM, both a significant IMM term and a significant IMI term were considered an eQTL. Per gene, we called a maximum of one eQTL per chromosome. We called a *cis*-eQTL if the distance between the middle of the gene and the marker was <2 Mb, and a *trans-*eQTL otherwise. For all models, we also generated *P*-values by randomly distributing the gene expression values over the mpRILs for each gene. We used the *P*-values obtained by permutation to constrain the false discovery rate (FDR) and obtain significance thresholds. For thresholding the marker and interaction terms, we used the lowest *P*-value per gene per chromosome. For the developmental age variable, we used the *P*-values of the models with the lowest marker *P*-value per transcript per chromosome. All results are obtained using an FDR of 0.05 unless explicitly mentioned in the text. *P*-value thresholds are in Supplementary Table 1.

### Transcripts affected by development

Transcripts were considered significantly affected by the developmental process if they had a significant *P*-value for the development variable using the AAM, in all the 6 models (one per chromosome) that resulted in the lowest marker *P*-value.

### Calculating partial eta squared

We performed an anova on the model with the lowest marker *P*-value using the aov() function from the stats package and extracted from the effects vector the residual sum of squares (RSS) and the sum of squares explained by the marker (SSM). Note that the marker with the lowest *P*-value can differ between the SMM and the AAM. We then calculated the partial eta squared according to the following equation:


$$\frac{\mathrm{SSM}}{\mathrm{SSM}+\mathrm{RSS}}$$

### GO enrichment

We performed gene ontology (GO)-term (Ashburner et al. 2000; Davis et al. 2022; The Gene Ontology Consortium *et al*. 2023) enrichment on genes that map to specific hotspots. We tested for enrichment of GO terms from the subcategories biological process, molecular function, and cellular component using a hypergeometric test. As the total gene set, we used the genes on the 12,029 transcripts used in the eQTL mapping. We called significant enrichment if the *P*-value < 0.001 and at least 3 genes were associated with this GO-term.

## Results

### Developmental age estimation by PCA

We quantified the developmental age of the mpRIL by performing a PCA on the normalized gene expression counts. Estimating relative differences in developmental age using a PCA was shown to be effective in a previous study (Jovic *et al.* 2019). PC1 explained ∼48% of the gene expression variation and PC2 explained ∼5% (Fig. 1a). Under the assumption that the developmental age would be the largest contributor to the variation in gene expression, we investigated whether PC1 is a good proxy for the developmental age of the mpRILs. First, we looked at the expression of the yolk protein *vit-2* gene, since it is part of a cluster of 53 genes (developmental indicator genes) that show a robust linear increase in expression during the L4 stage (cluster 1 in the study by Snoek *et al.* 2014, see figure S4 for gene names). The expression of *vit-2* increases as the projection of the mpRILs on PC1 increases (Pearson correlation ∼0.91; Fig. 1a). Second, we investigated whether the other developmental indicator genes also show a positive correlation with PC1. We found a strong positive correlation between the projection on PC1 and the mean expression of developmental indicator genes (Pearson correlation ∼0.97; Fig. 1b). The association between the developmental indicator genes and PC1 is further verified by high projections of these genes on PC1 (Supplementary Fig. 1). We validated the developmental indicator gene approach by taking a larger set of 2,050 genes shown to be monotonically rising in a different study (Hendriks *et al.* 2014). The mpRILs ordered by their projection on PC1 sort the expression of these genes well (Supplementary Fig. 2). Third, we verified the estimated developmental ages of the mpRILs by using the RAPToR package (Bulteau and Francesconi 2022). The RAPToR package estimates the age of an organism based on its expression profile, by using an appropriate transcriptomic time series as a reference. The high correlation between the RAPToR ages and PC1 (Pearson correlation ∼0.91) confirms that PC1 can be used as a proxy for the developmental age. However, the mpRILs ordered by the RAPToR age estimates provided a sorting that was less consistent with the known monotonically increasing expression of the developmental indicator genes (Supplementary Fig. 3) compared with PC1 (Supplementary Fig. 4). Hence, we used the projections of the mpRILs on PC1 as our developmental age estimates in the subsequent analysis. Note that PC1 appears to be associated with the genetic background, given the nearly identical loadings of the parental duplicates on PC1 (Supplementary Fig. 5). We conclude that PC1 is strongly associated with the developmental process and can be used to approximate the developmental age of the mpRILs.

### The effect of developmental age variation on eQTL mapping

To study the effect of variation in developmental age on eQTL mapping, we compared a linear SMM with 2 models that include the developmental age as a cofactor: (1) the linear AAM and (2) the linear IM. For the IM, we distinguished eQTLs due to a significant marker effect from eQTLs and a significant interaction between the marker and the developmental age. While the IM, marker (IMM) term, detects additive differences in gene expression, a significant IM, interaction (IMI) term, indicates an eQTL that influences the slope of gene expression over the developmental age. While the eQTLs detected by the models partially overlap, each model also detects a subset of unique eQTLs (Supplementary Fig. 6). We focus our initial investigation mostly on the SMM and AAM. We refer to SMM-only eQTLs, AAM-only eQTLs, or SMM + AAM eQTLs to distinguish between eQTLs found by only the SMM, AAM, or both, respectively. We first show the difference between the models in terms of the distribution of eQTLs over the genome. Next, we show the quantitative effect of dynamical eQTL mapping by comparing the SMM with the AAM in terms of the number of eQTLs, *P*-values, strength of the marker effects, and heritability of transcript levels. Finally, we discuss the application of the IM for dynamic eQTL mapping.

#### Developmental age variation affects the manifestation of eQTL hotspots

**Fig. 2.**
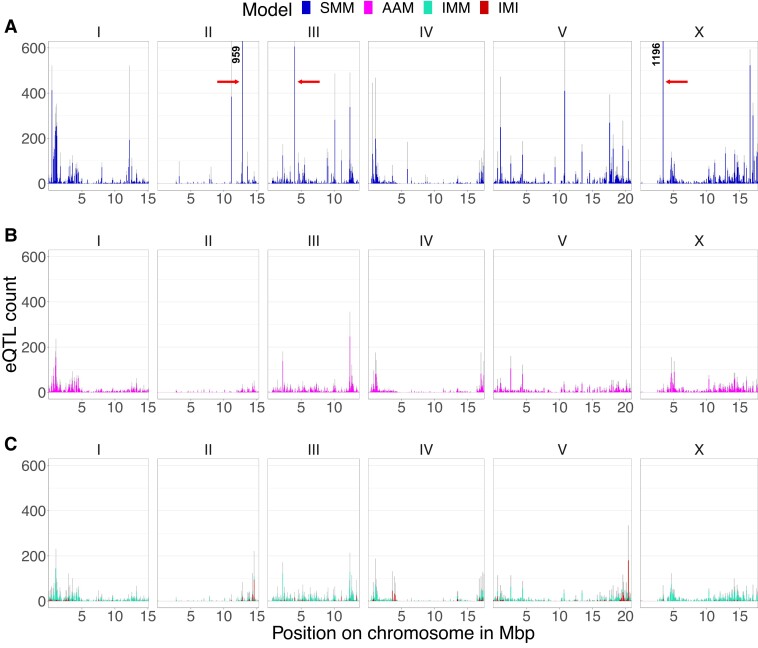
Distribution of eQTLs over the chromosome according to the different models. *X*-axis shows the position of the genome. *Y*-axis shows the number of eQTLs that map to this position. a) SMM. Arrows indicate the 3 largest hotspots. b) AAM. c) IM. Cyan and red show eQTLs according to the IMM and IMI, respectively. Counts are in terms of the number of transcripts mapping to the location.

We investigated whether the AAM shows an altered distribution of eQTLs over the genome compared with the SMM. The SMM yielded several hotspots enriched in *trans-*eQTLs, whereas *cis-*eQTLs are distributed evenly over the genome (Fig. 2a; Supplementary Fig. 7a). The AAM yielded fewer eQTLs, with a particularly strong decrease in the number of large hotspots (Fig. 2b; Supplementary Fig. 7b) and *trans*-eQTLs in general (Table 1). On the other hand, the number of (unique) markers with an eQTL increased (Supplementary Table 2). The largest hotspot found by the SMM, located at position 3.4 Mb on chromosome X, significantly affects the expression of 1,196 transcripts. In contrast, the AAM yielded only a single eQTL at this location. Replacing PC1 with the mean developmental indicator gene expression as a cofactor in the AAM gave a very similar eQTL distribution (Supplementary Fig. 8), with the same disappearing hotspots. These results indicate that most hotspots are caused by developmental variation.

**Table 1.** Significant effects detected by the single marker, additive age, and IMs.

	Single marker model (SMM)	Additive age model (AAM)	Interaction model, marker term (IMM)	Interaction model, interaction term (IMI)
eQTLs detected (FDR <0.05)	18,605	8,666	6,886	1,161
*Cis*/Local	2,136	1,954	1,814	92
*Trans*/Distant	16,469	6,712	5,072	1,069
*Cis*/*Trans* ratio	0.13	0.29	0.36	0.08
Transcripts w/eQTL	8,875	6,133	5,180	904
eQTLs detected (FDR <0.1)	27,445	13,369	10,938	4,276
Transcripts affected by developmental process	—	9,473	8,806*^a^*	8,806*^a^*

An FDR of 0.05 is used unless otherwise specified.

^*a*^Applies to additive developmental age term of IM.

We hypothesized that the SMM-only hotspots are developmental QTLs. Under this hypothesis, we would expect that (1) transcripts involved in developmental processes are enriched at the hotspots that disappear when using the developmental age as a cofactor, (2) the genotype at the hotspot position is linked to the developmental age, and (3) SMM-only eQTLs primarily map to such hotspots.

First, we investigated whether genes mapping to the largest hotspot were enriched for developmental GO terms. The genes having an eQTL at the hotspot position were enriched for developmental processes such as *oocyte maturation*, *polar body extrusion after meiotic division*, *P granule*, *eggshell formation*, *pseudopodium*, *amoeboid sperm motility*, *male meiosis chromosome segregation*, *mitotic spindle pole*, and *structural constituent of cuticle*. This supports the involvement of this hotspot locus in development. Other hotspots were similarly enriched for genes associated with developmental processes (Supplementary File 1). Apart from functional categories, hotspots also share many of the same genes. As an example, more than 80% (896 out of 1,115) of the transcripts that map to the hotspot at position 12.8 Mb of chromosome II also map to the hotspot at position 3.4 Mb of chromosome X. This paints a picture of genes involved in developmental processes indiscriminately mapping to hotspots due to a shared association with the developmental age.

**Fig. 3.**
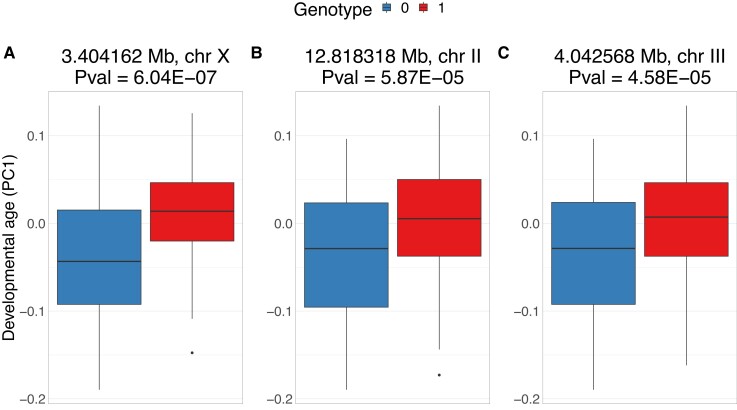
Distribution of developmental ages per allele at the 3 largest hotspots. Hotspots are indicated in Fig. 7 by red arrows. *P*-Values are obtained with a single marker linear model of PC1 ∼ marker genotype (see Supplementary Fig. 9).

Second, we investigated whether the markers at the hotspots that are only found with the SMM are linked to the developmental age by comparing the distributions of the developmental age between the alleles of 3 major hotspot loci (indicated by red arrows in Figs. 2 and 3). As expected, the distribution of developmental ages was clearly different between the genotypes at these hotspots. These loci could be linked to the developmental age because they regulate or affect the developmental speed. In this view, SMM-only hotspots affect gene expression through their effect on the developmental process itself. To investigate this, we ran SMMs with the developmental age as a trait. In line with our hypothesis, the marker of the largest hotspot was also the most predictive of the developmental age of the mpRILs (Supplementary Fig. 9).

**Fig. 4.**
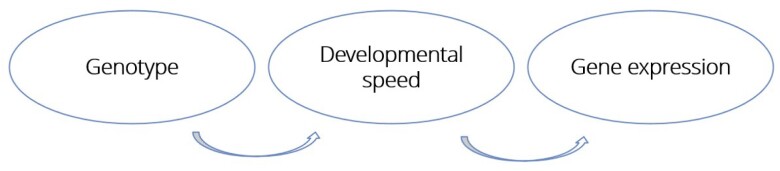
Schematic of how some hotspots cause gene expression variation. Some hotspots influence the developmental speed, causing the transcriptional program of the genotypes to be at distinct developmental ages at a given moment in chronological time. This in turn causes expression variation in the large number of transcripts whose expression level depends on the developmental age. The developmental age is the mediator through which these loci affect gene expression.

Third, we investigated the distribution of SMM-only eQTLs (Supplementary Fig. 10). In line with our hypothesis, this subset of eQTLs maps primarily to the disappearing hotspots. Together these 3 results suggest that most hotspots can be attributed to genes whose expression changes based on the developmental age mapping to loci that influence the developmental speed (Fig. 4).

#### Developmental age as a cofactor strongly affects the quantitative eQTL landscape

Having observed large differences between the models in the distribution of eQTLs over the genome, we further investigated the quantitative effect of adding the developmental age as a cofactor on the number of eQTLs and the *P*-values and allelic effects of individual eQTLs. First, we examined the impact of the developmental process on the transcriptome using the developmental age term of the AAM. We find 9,473 transcripts (out of 12,029 polycistronic transcripts) with expression levels significantly affected by the developmental age [−log(*P*) > 1.79] (FDR = 0.05; Table 1). This number is in line with previous reports on the N2 reference strain (Snoek *et al.* 2014) and emphasizes the substantial changes in overall gene expression during development.

**Fig. 5.**
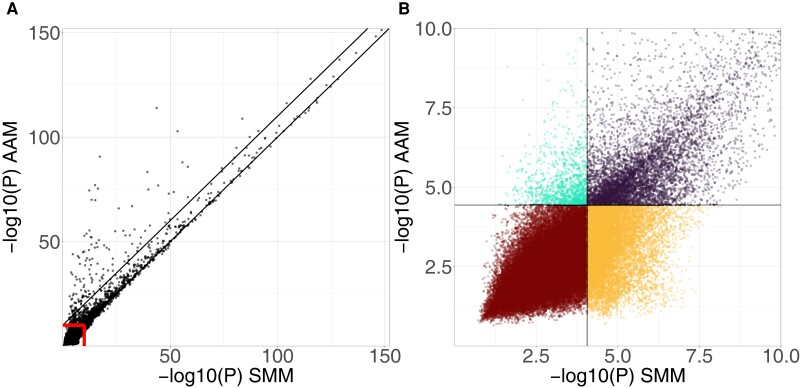
Comparison *P*-values obtained with the SMM and AAM. a) −log_10_(*P*) of SMM vs AAM, i.e. higher value corresponds to a lower *P*-value. The *x*-axis shows the lowest marker *P*-value per chromosome per transcript obtained with the SMM (72,174 total plotted *P*-values). The *y*-axis shows the same for the AAM. Since we call a maximum of one eQTL per transcript per chromosome, all eQTLs are represented in the plot. The red lines show the subsection of the plot depicted in b). The lower black line is the identity line, such that eQTLs above the line have a lower *P*-value according to the AAM, whereas eQTLs below the line have a lower *P*-value according to the SMM. eQTLs above the top black line are strong developmental effect eQTLs (*P*-value 10 orders of magnitude lower with AAM when compared with SMM). b) Zoomed-in subsection of a). Points are colored by whether the eQTL was detected with none (brown), both (purple), or one of the models (orange for SMM-only eQTLs and cyan for AAM-only eQTLs).

**Fig. 6.**
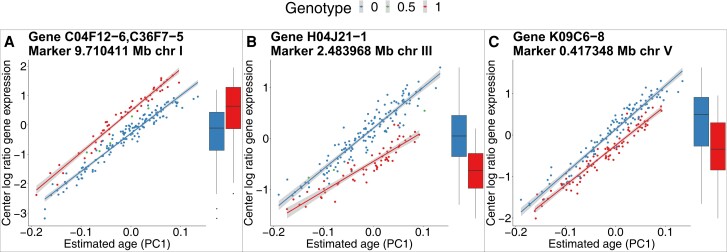
Gene expression of 3 examples of strong developmental effect eQTLs. Line plots show the CLR of fpkm values (*y*-axis) over the developmental age (*x*-axis) for 3 examples of strong developmental effect eQTLs. Colors correspond to the genotype at the eQTL position. Boxplots show the magnitude of gene expression without the context of the developmental age.

Next, we compared the *P*-values of the marker effects between the SMM and AAM (Fig. 5a). For a subset of eQTLs (151), the *P*-value was more than 10 orders of magnitude lower in the AAM compared with the SMM [*Strong developmental effect eQTLs*, above top black line in (Fig. 5a)]. The transcripts with such eQTLs all displayed a gene expression pattern over the developmental age that has a clear linearly increasing trend and little within allele variation compared with between allele variation (Fig. 6). Most strong developmental effect eQTLs are detected by both the SMM and AAM. Examining the changes in *P*-value around the thresholds (Fig. 5b; Supplementary Table 1) revealed that many eQTLs were only detected by one of the models. eQTLs that are differentially detected between the models are of special interest as in these cases the qualitative result of the eQTL mapping procedure is impacted, potentially obfuscating or revealing relevant biology.

The total number of eQTLs decreased by ∼53% between the SMM (18,605) and the AAM [(8,666) Table 1]. The AAM detected 1,221 AAM-only eQTLs that would not be detected by the SMM (Fig. 5b, cyan color; Supplementary Table 2). On the other hand, 11,160 SMM-only eQTLs were no longer significantly associated with gene expression when the AAM is applied (Fig. 5b, orange color). In conclusion, adding the developmental age as a cofactor to the model changed the detection outcome for more than 12,000 potential eQTLs.

#### Dynamical eQTL mapping can reveal additional regulatory effects by correcting for developmental age

**Fig. 7.**
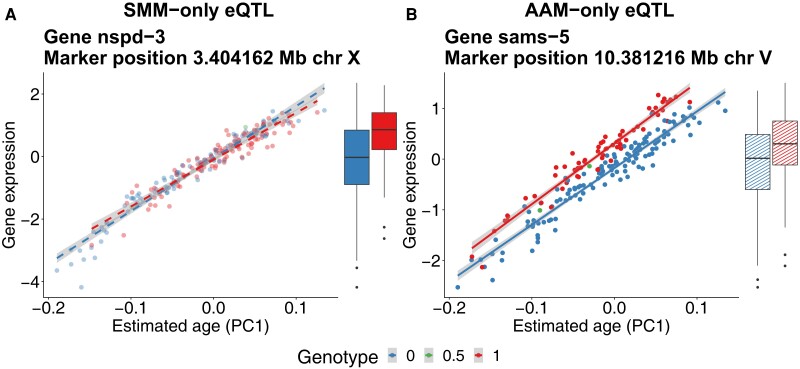
Examples of differentially detected eQTLs. Dashed line and dashed boxplot indicate no significant difference between alleles. a) SMM-only eQTL affecting the nspd-3 gene. The SMM only considers mean gene expression differences between alleles (solid boxplot). For SMM-only eQTLs such mean differences occur because one genotype has on average a lower developmental age than the other. In this case, the blue genotype is on average developmentally younger. The alleles shown correspond to the hotspot locus on ∼3.4 Mb on chromosome X whose developmental age distribution is depicted in Fig. 3a. For SMM-only eQTLs correcting for the developmental age reveals there is no significant difference in gene expression at any point along the developmental trajectory of the mpRILs (dashed lines). b) AAM-only eQTL affecting the sams-5 gene. For the AAM-only eQTLs, there is no significant mean gene expression difference between the alleles (dashed boxplot). However, when considering the context of developmental age differences, a clear difference between the genotypes is revealed (solid lines).

To understand why some eQTLs are differentially detected between the SMM and AAM, we looked at individual eQTLs. For the SMM-only eQTLs, the observed significant difference in gene expression between the alleles (Fig. 7a, boxplot) can be explained primarily by the difference in developmental age (Fig. 7a, line plot; Fig. 3a). The blue genotype is on average developmentally younger than the red genotype, causing gene expression differences. For this subset of eQTLs, accounting for developmental variation in the model shows that the locus is not significantly associated with gene expression when stratified for developmental age (Fig. 7a, line plot). For the AAM-only eQTLs, there is no significant mean difference in gene expression between the alleles (Fig. 7b, boxplot). However, the AAM reveals a significant association, over the entire range of developmental ages of the mpRILs, between this locus and gene expression (Fig. 7b, line plot). In both cases of differentially detected eQTLs, the SMM is being confounded by an association between the marker and the developmental age. These examples show how dynamical eQTL mapping can control for developmental variation in the mapping population (Fig. 7a) and uncover regulatory regions obscured by this developmental variation (Fig. 7b) on a gene-by-gene basis.

#### Developmental age variation impacts the allelic effect of eQTLs

**Fig. 8.**
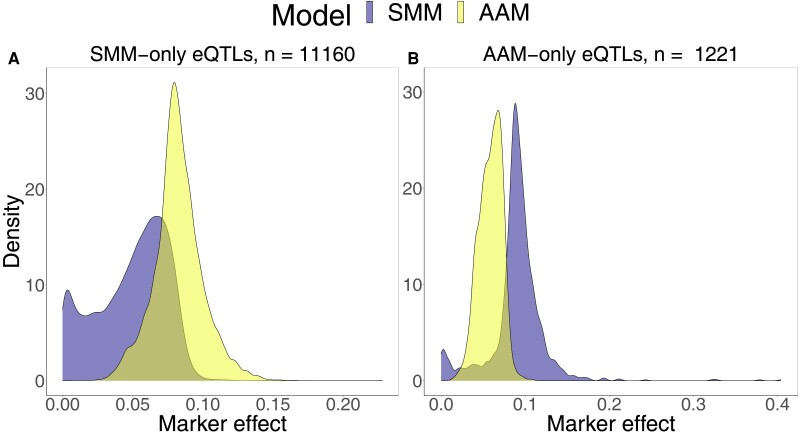
Density plot of the partial eta squared of marker effects for subsets of eQTLs according to the SMM and AAM. For the eQTLs in the subset, we selected the marker with the lowest *P*-value according to each of the models and calculated the partial eta squared of the marker effect using the respective model: a) SMM-only eQTLs, b) AAM-only eQTLs.

The estimated allelic effects are expected to differ between the SMM and AAM, given the large number of transcripts affected by development. In the previous section, we showed that for SMM-only eQTLs, variation in mean gene expression between alleles does not always imply gene expression differences at a specific point in development (Fig. 7a). In such cases, we expect the allelic effect on gene expression estimated by the SMM, which considers only mean differences between alleles, to be higher than the allelic effect estimated by the AAM, which accounts for the developmental age. Conversely, for AAM-only eQTLs, the allelic effect can be obscured unless viewed over a developmental axis (Fig. 7b; Supplementary Fig. 11). In this scenario, we expect the allelic effect estimated by the AAM to be higher than the SMM estimation. To quantify the strength of the allelic effect, we calculated the (partial) eta squared of the marker variable for SMM-only and AAM-only eQTLs. Indeed, the SMM tends to infer a stronger allelic effect for SMM-only eQTLs (Fig. 8a), whereas the AAM tends to infer a stronger allelic effect for AAM-only eQTLs (Fig. 8b). We also investigated the effect of adding developmental age as a cofactor in models of the narrow sense heritability (*h*_2_). We find that with developmental age as a cofactor the *h*_2_ of gene expression changes by more than 0.05 for ∼47% of genes and that the direction of change depends on the type of eQTL(s) (SMM–eQTL and/or AAM–eQTL) mapping to that gene (Supplementary Text 1). These findings indicate that the allelic effect of eQTLs and the *h*_2_ of gene expression can be either obscured or exaggerated by hidden developmental variation.

### Interactions between the genomic background and developmental age affect the dynamics of gene expression

**Fig. 9.**
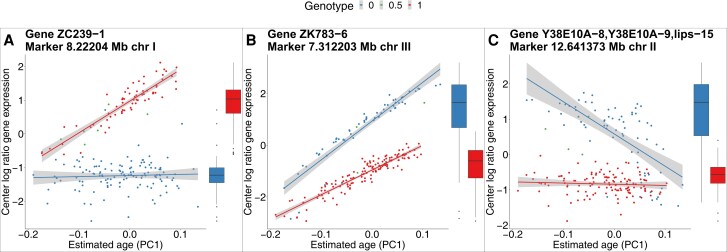
A selection of representative eQTLs detected with IMI term. Colors correspond to alleles. Lines are the best fit of the IM model (gray area is 95% CI) to gene expression over developmental age. Boxplots show the magnitude of gene expression without the context of the developmental age. a) eQTL that, depending on genotype, causes either increasing gene expression as development progresses or a constant expression level. b) eQTL that causes the slope of gene expression over developmental age to be higher for one genotype compared with the other. c) eQTL for which one genotype exhibits negative regulation of gene expression while the other genotype shows constant gene expression.

Genetic loci can affect, apart from the magnitude and timing of gene expression, also the shape of the dynamical pattern (Francesconi and Lehner 2014). To investigate the frequency of interactions between marker effects and the developmental age, we applied a linear IM for eQTL mapping. A significant interaction term in this model indicates different dynamics between the marker alleles (Fig. 9). Using the IM results in a ∼20% decrease in the number of marker effects detected (6,886) compared with the AAM. On top of the marker effects, the IM detects a significant interaction between the marker and developmental age for 1,161 transcripts (FDR = 0.05 Table 1; Fig. 9; FDR = 0.1 shown in Supplementary Fig. 12). Investigating the distribution of interaction eQTLs over the genome shows that these also cluster in hotspots. The hotspot with the most eQTLs (20.440891 Mb, chr V) affects the expression of almost 200 transcripts. Despite the correction for the developmental age inherent to the model, the transcripts mapping to this hotspot are significantly enriched in GO-terms associated with the cell cycle, meiosis, mitosis, and other developmental processes (Supplementary File 2). This locus could therefore be an important determinant in the developmental speed during the range of developmental ages spanned by the mpRILs by affecting the rate of up- or downregulation of many transcripts involved in developmental processes (Supplementary Fig. 13a–c). The genotypes corresponding to this marker also differ substantially in their distribution over the developmental age (Supplementary Fig. 13d), suggesting that its effect on the developmental speed was also present at younger developmental ages. This shows that the IM can detect loci that have large effects on the transcriptome by influencing the rate at which transcript levels change for many transcripts.

## Discussion

We have shown that eQTL hotspots that disappear after controlling for the developmental age can correspond to markers influencing the developmental speed. For such hotspots, there is likely no direct regulatory relationship between the hotspot and the genes with an eQTL mapping to it. After all, at any specific developmental age, there is no significant difference in expression between the alleles. Rather, one of the genotypes is on average further developed than the other, causing gene expression differences. Because the genes mapping to such hotspots are enriched for developmental functions, they are likely to have non-constant expression over the developmental age range of the mapping population. A non-zero slope of gene expression over the developmental age can cause differences in the overall magnitude of gene expression between marker alleles, even in the absence of gene expression differences between alleles at any specific developmental age, if developmental ages are not distributed equally between the alleles.

The mechanism through which a polymorphic SNP influences the developmental speed can be difficult to entangle because gene expression both drives and is the result of the developmental speed. Therefore, an eQTL might influence the developmental speed by affecting the expression of some genes, as a result changing the expression of many more genes. For some SNPs in hotspots, the association with the developmental age can be understood from the genomic context. For example, the large hotspot at the beginning of chromosome X maps close to the *vit-5* gene encoding for the Vitogellin-5 protein which is a major yolk component. Inhibition of *vit-5* by means of RNAi has been shown to result in slower rates of post-embryonic growth (Kamath *et al.* 2003). Interestingly, the maternal expression of vitellogenin genes appears to be a major determinant of developmental speed (Perez *et al.* 2017). As a second example, the hotspot at 10.1 MB on chromosome III is located in the *kel-10* gene. The human ortholog (*KLHL10*) of this gene is involved in spermatogenesis, and *kel-10* is affected by the *daf-2* gene, a well-known regulator of lifespan in *C. elegans* (Kenyon *et al.* 1993).

Hotspots are often assumed to be global regulators of gene expression, although it is well known that hotspots can also result from genes with correlated expression because of uncontrolled latent factors (Breitling *et al.* 2008; Michaelson *et al.* 2009). We show that developmental variation can be a major cause of such correlations, to the extent that most prominent hotspots are no longer present after correcting for the developmental age. Therefore, we posit that the default interpretation for hotspots should be an association between the hotspot locus and the developmental age.

Loci that influence the developmental speed need not be seen as merely a confounder in eQTL mapping. Instead, they are crucial determinants of the expression state of an organism. Investigating the eQTLs detected by a non-dynamical but not a dynamical model is a convenient approach to search for loci that are causative for developmental age variation. This would allow a distinction between loci that cause gene expression differences even if organisms are at the same developmental age (eQTLs detected by AAM) and loci that affect gene expression by causing developmental age differences (SMM-only eQTLs). This approach is not limited to the developmental process but can be applied to any process which leaves a sufficiently strong signal in transcriptomics data. More generally, including latent factors in eQTL mapping can allow one to distinguish between loci that influence gene expression through their effect on generic processes and other effects of the genetic background on gene expression.

Because eQTLs can also cause allele-dependent non-linear expression patterns over the developmental age (Francesconi and Lehner 2014), we performed the eQTL mapping using a natural spline of the developmental age (Supplementary Text 2). Using the interaction term of this model, we hoped to identify eQTLs responsible for complex dynamical patterns. However, constraining the FDR of the natural spline model using the same permutation strategy, we used for the linear models was not possible, as the *P*-values obtained on the actual dataset were barely distinguishable from the *P*-values obtained on the randomized dataset. We suspect this is in part due to a lack of statistical power, resulting from a combination of the size of the population (199 lines) and the dense genetic map (8,933 SNPs) used for the mapping. An additional factor could be that our population has a narrow distribution of developmental ages. A study by Francesconi and Lehner (2014) detected many non-linear patterns using natural splines. The population used in their study contained worms in the L3, L4, and young adult developmental stages. The mpRILs used in this study were all in the L4 larval stage (Supplementary Fig. 14). Possibly, non-linear dynamics only occur over a wider range of developmental ages than is covered by the mpRILs in our study or when multiple distinct stages are present. To investigate, we clustered 2,346 transcripts whose expression oscillates with a period of ∼8 h (Hendriks *et al.* 2014) using k-means. The clustering revealed mostly monotonic or constant gene expression patterns for the clusters (Supplementary Fig. 15). This indicates that the developmental range spanned by the mpRILs is not broad enough to clearly observe the oscillations to which a large part of the *C. elegans* transcriptome is subject (Kim *et al.* 2013; Hendriks *et al.* 2014; Meeuse *et al.* 2020). Therefore, the natural spline model may not be suitable or, at least superfluous, for mapping eQTLs in our experiment.

In this paper, we used a relatively simple method (PCA) to quantify the developmental age. Various more complex methods that extract developmental ages from transcriptomics data have already been developed. An example is the RAPToR framework, which projects the samples on an interpolated reference time series (Bulteau and Francesconi 2022). Because we wanted a method that was generally applicable, we did not want to be completely dependent on a reference (time) series. Our approach enables the detection of other (semi-)linear processes affecting gene expression on a genome-wide scale and can be applied to other published eQTL studies in *C. elegans* (Li *et al.* 2006, 2010; Snoek *et al.* 2017; Sterken *et al.* 2017, 2020; van Wijk *et al.* 2023) and beyond (Ben-David *et al.* 2021). Furthermore, our method, despite being simpler, provided an ordering of the expression of developmental indicator genes that was more consistent with the monotonic linear increase described in previous literature.

We showed that PC1 accounted for most of the developmental variation in our population. The study by Francesconi and Lehner showed that the developmental signature was distributed between PC1 and PC2, which were assigned the interpretation of oogenesis and spermatogenesis, respectively (Francesconi and Lehner 2014). Together, PC1 and PC2 explained ∼50% of the gene expression variation, which is close to our PC1 (∼48%). In accordance with 2 developmental axes, the projected samples formed an almost circular trajectory on PC1 and PC2. The authors showed that, while the distribution of developmental ages is centered on the L4 larval stage, the population also includes individuals from late L3 and early adult. It is possible that transitions between developmental stages cause developmental variation in gene expression to be distributed over more than one PC axis.

A previous study scored the time to first egg phenotype of the mpRILs (Snoek *et al.* 2019). Because time to first egg is a clear indicator of the developmental speed, we hypothesized that this phenotype would correlate with PC1. Surprisingly, there was almost no correlation between PC1 and the time to the first egg, or indeed any of the other phenotypes scored in this study (Supplementary Fig. 16). This can likely be partially attributed to experiment variation. A second relevant factor could be the reported inverse relationship between the duration of larval development and the time between the first adult molt and the development of the first embryo (Perez *et al.* 2017). Third, the developmental speed could differ between developmental stages. Development is a complex process with crucial developmental events likely being regulated by independent timers (Filina *et al.* 2022). Accordingly, correlations between the durations of 2 larval stages in experiments with genetically identical worms are low when temperature is strictly controlled (Mata-Cabana *et al.* 2022). On top of this, the genetic background can have stage-specific effects on developmental speed (Filina *et al.* 2022; Mata-Cabana *et al.* 2022). Stage-specific developmental variation would de-correlate developmental ages over the course of chronological time. Verifying this third explanation would require an experiment in which samples are obtained within and between time points. Such an experiment would allow us to measure the degree to which developmental variation is maintained on various timescales. More importantly, this experiment would allow us to compare gene expression over developmental age with gene expression over chronological age. In this way, we could definitively show whether gene expression variation over PC1 is due to developmental variation. Furthermore, it would allow for an investigation into the extent to which eQTL patterns over developmental time translate to eQTL patterns over chronological time.

In conclusion, we have shown that performing a PCA and including PCs into the model for eQTL mapping leads to an improved understanding of eQTL patterns by separating the effects of generic processes, such as development, from other regulatory effects of the genetic background. Furthermore, the effect of these generic processes on gene expression and their interaction with the genetic background can be understood by comparing models with and without PCs. Within an experimental condition, PCs can correspond to the developmental process, technical noise, or other latent variables unrelated to the research question. Including PCs as representations of such latent variables in the mapping procedure is a convenient way to control for or come to understand sources of variance confounding the effect of interest (O’Duibhir *et al.* 2014). We recommend that this should be standard practice in eQTL mapping studies given the large effect of processes like development on the transcriptional state of the cell.

## Supplementary Material

jkad273_Supplementary_Data

## Data Availability

All code used in the study is available at github.com/SnoekLab/van-Eijnatten-etal-2023. The genetic map used for eQTL mapping can be found in additional file 1 of the original paper on the multi-parental population (Snoek *et al.* 2019). Raw RNA-sequencing data used for constructing the genetic map and eQTL mapping are available from the NCBI Sequence Read Archive database (SRA; accession number PRJNA495983; www.ncbi.nlm.nih.gov/bioproject/PRJNA495983/). Normalized read counts and *P*-value matrixes of the eQTL mapping for each of the models can be found on WormQTL2 (Snoek *et al.* 2020) as Eijnatten_etal_2023 SMM/AAM/IMM/IMI. Supplemental material available at G3 online.
