# Supplementary material for: The effect of developmental variation on expression QTLs in a multi parental Caenorhabditis elegans population
Source: G3 (Bethesda). 2023 Nov 28;14(2):jkad273. doi: 10.1093/g3journal/jkad273 (PMC10849341; doi:10.1093/g3journal/jkad273)
Supplement: jkad273_Supplementary_Data [file jkad273_supplementary_data.zip › Supplemental_Tables_and_Figures_G3-2023-404548.docx]

**Supplementary tables**

**Table S1:** –log10 p-value thresholds linear models.

|  | Single marker model | Additive age model | | Interaction model | | |
| --- | --- | --- | --- | --- | --- | --- |
| FDR | Marker | Marker | Developmental age | Marker | Developmental age | Interaction |
| 0.05 | 4.06 | 4.44 | 1.79 | 4.60 | 3.07 | 5.60 |
| 0.1 | 3.52 | 3.87 | 1.43 | 3.98 | 2.65 | 4.52 |

**Table S2: Statistics on quadrants, denoted by different colors in figure 5B.** The first row describes the number of gene/marker combinations per quadrant, the second and third row show the number of unique markers per quadrant for the SMM and AAM respectively and the fourth row displays the number of eQTLs per quadrant for which the location of the best marker changes by at least 1MB between the SMM and AAM.

|  | Brown quadrant | Orange quadrant | Cyan quadrant | Purple quadrant |
| --- | --- | --- | --- | --- |
| Total gene/marker combination | 52348 | 11160 | 1221 | 7445 |
| Unique markers SMM | - | 1120 | 474 | 2364 |
| Unique markers AAM | - | 2300 | 676 | 2726 |
| Number of markers switching between models | - | 5306 | 667 | 1576 |

**Supplementary figures**


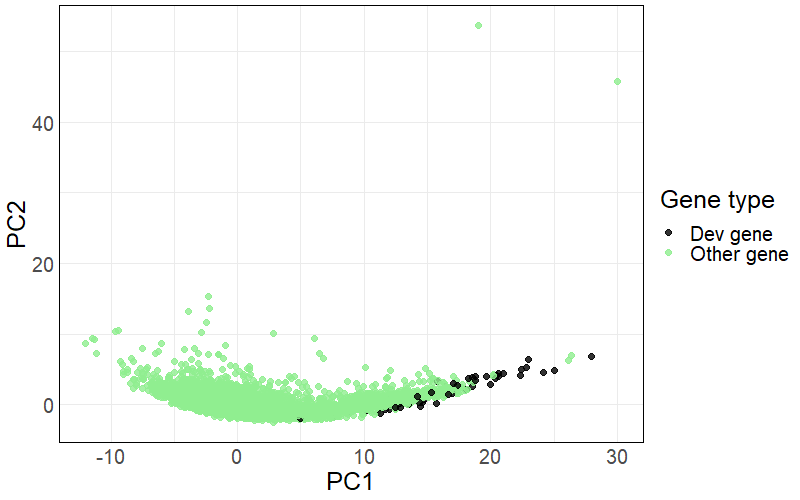


**Figure S1: Developmental indicator genes have high scores on PC1.** Projections of all 12029 genes used in the mapping on PC1 (x-axis) and PC2 (y-axis). Black color denotes the 53 developmental indicator genes, while the green color denotes all other genes.


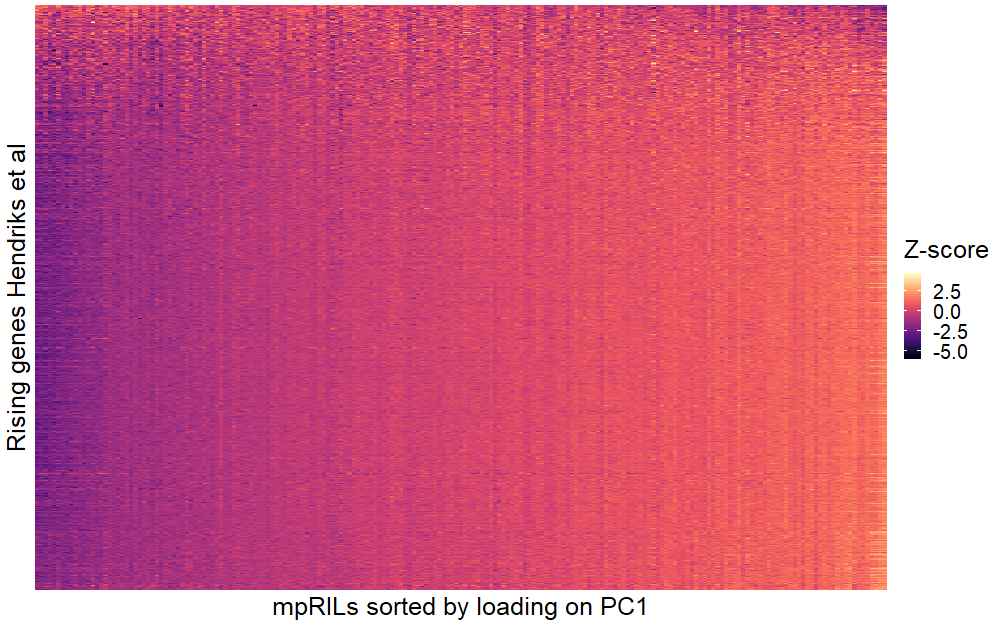


**Figure S2: Heatmap of expression monotonically rising genes.** Heatmap of z-score of the center log ratio of gene expression of 2050 genes that were identified as monotonically increasing between the beginning of the L3 stage and the young adult stage(Hendriks et al. 2014). The columns are ordered by the projection of the mpRILs on PC1 and the rows are ordered from low to high by the slope of the expression values over PC1. Slopes were calculated as the coefficient of the developmental age variable in a linear model of gene expression ~ PC1.


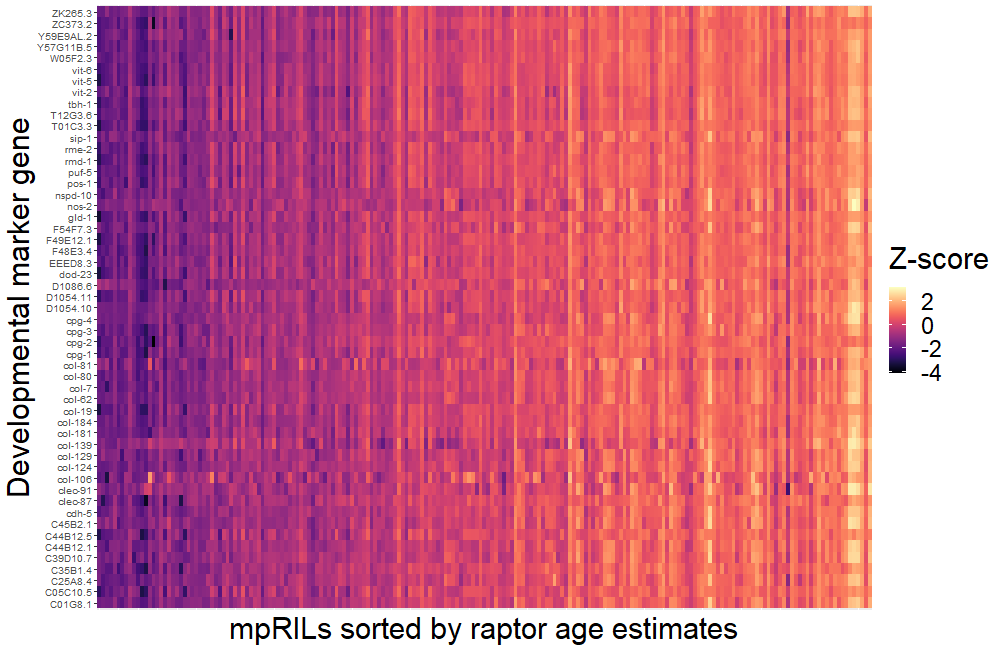


**Figure S3: Expression of developmental indicator genes of mpRILs ordered by RAPToR age estimates.** Heatmap of z-score of the center log ratio of gene expression of developmental indicator genes (y-axis) for the mpRILs sorted by the RAPToR age estimates (x-axis).


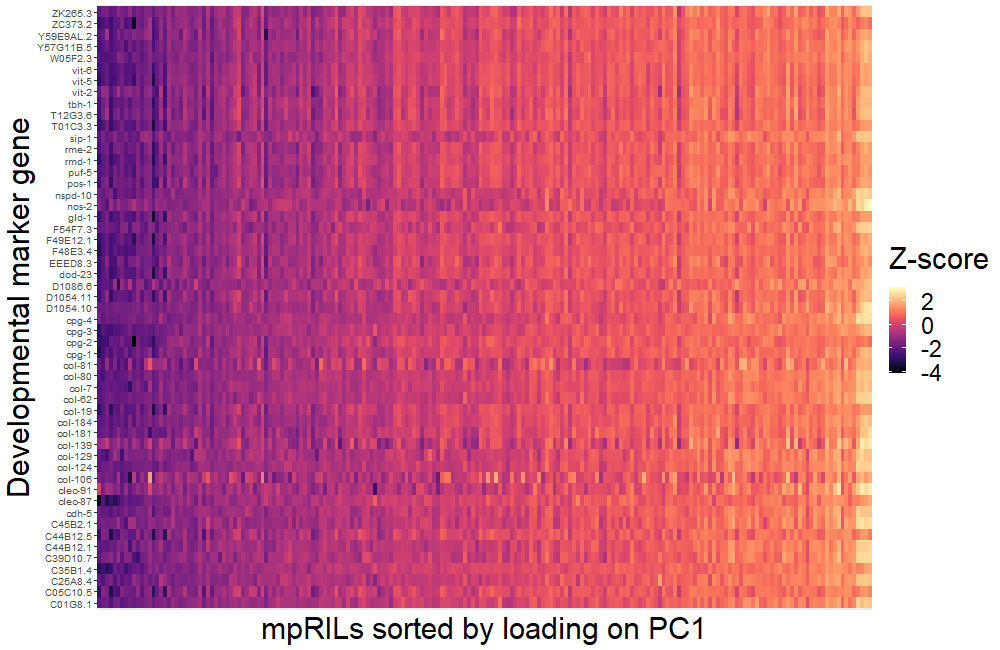


**Figure S4: Expression of developmental indicator genes of mpRILs ordered by PC1.** Z-score of center log ratio of gene expression of developmental indicator genes (y-axis) plotted for the mpRILs sorted by their projection on PC1 (x-axis).


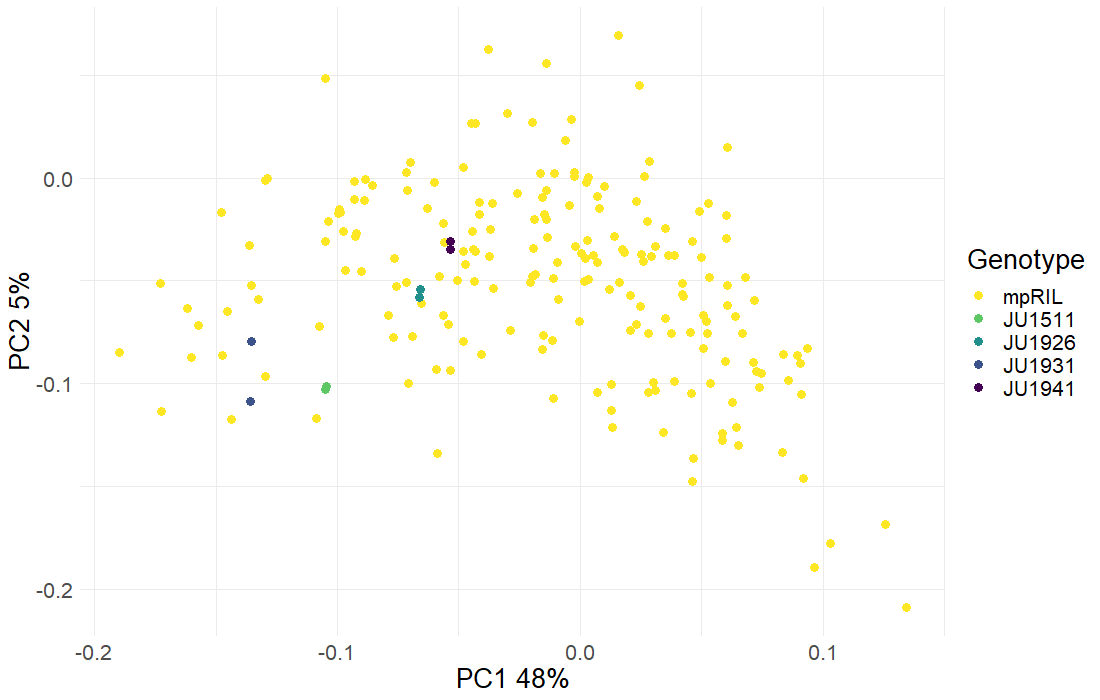


**Figure S5: Parental replicates have almost identical projections on PC1.** Projection of population on the first two principal components, colored by whether the worm corresponds to a parental genotype or one of the mpRILs.


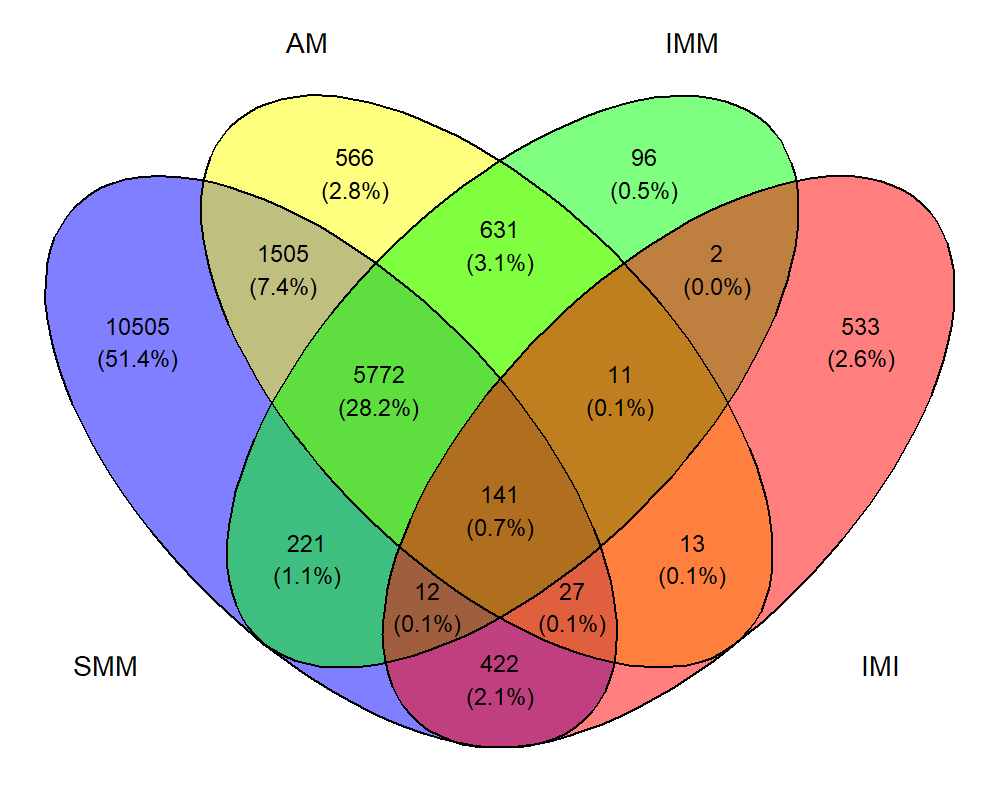


**Figure S6: Venn diagram of eQTL overlap between various models.** Overlap between the single marker model (SMM, blue), additive age model (AAM, yellow), interaction model, marker term (IMM, green) and interaction model, interaction term (IMI, red). eQTLs are overlapping between models if the models detect a significant marker on the same chromosome for the same gene. Percentages are in terms of the union of the eQTLs detected by the four models.


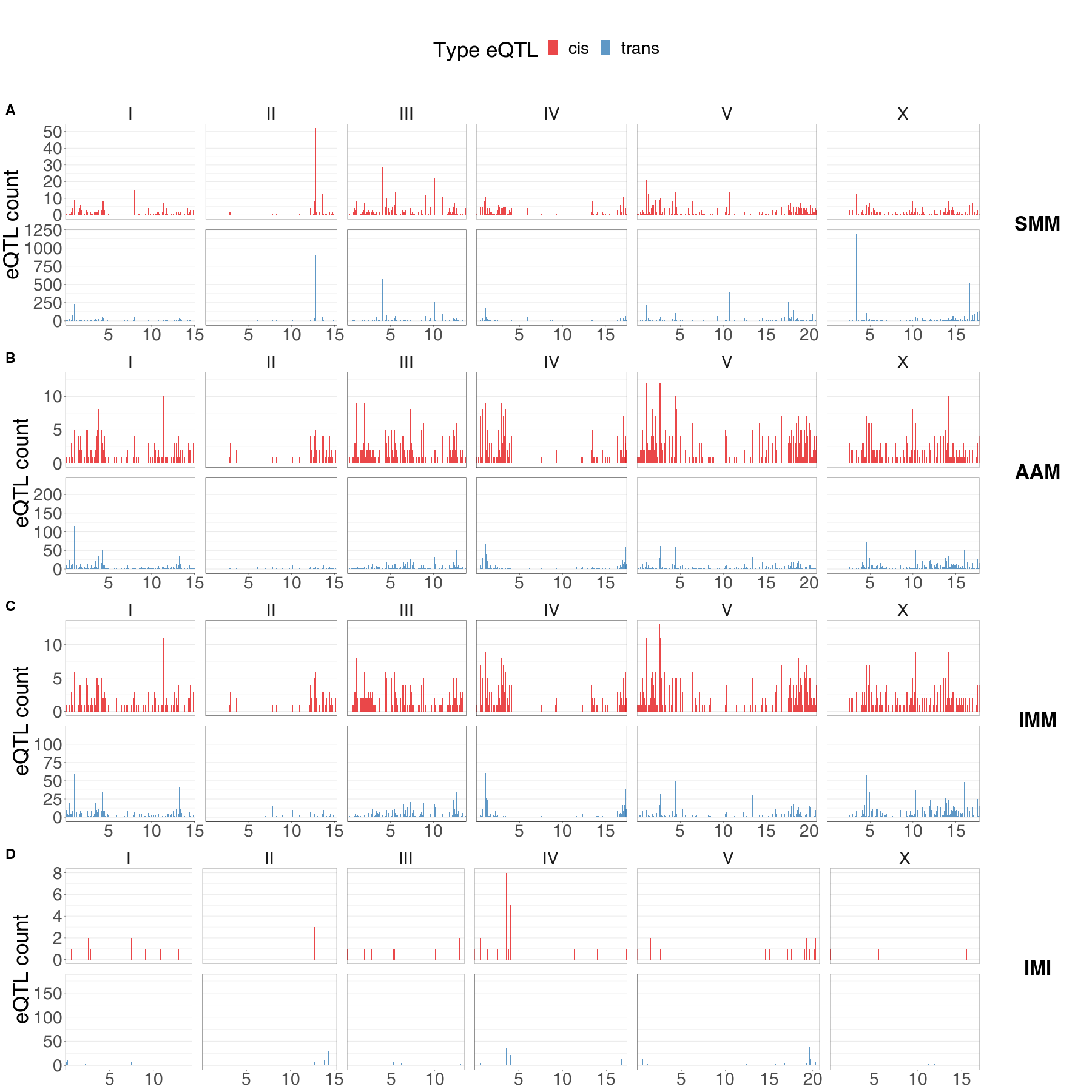
**Figure S7** ***Cis*/*trans* distribution of eQTLs over the genome.** Blue and red colors indicate *trans-e*QTLs and *cis*-eQTLs respectively. Note that the range of the y-axis differs between and within the sub figures.


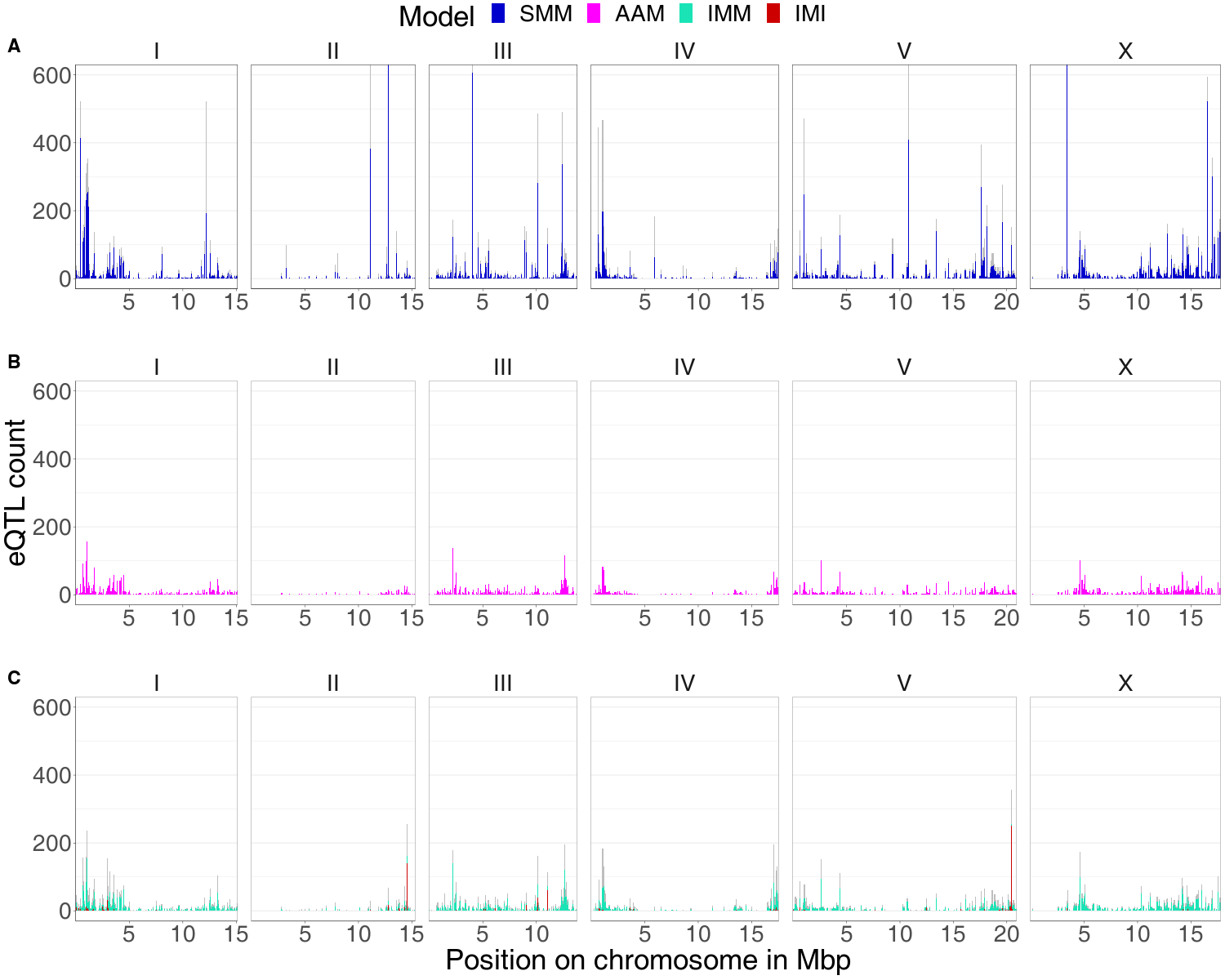
**Figure S8: Distribution of eQTLs when using the mean developmental indicator gene expression as a co-factor instead of PC1.** Compare to figure 2. X-axis shows the position on the genome. Y-axis shows the number of eQTLs that map to this position. **A)** SMM but with mean developmental gene expression as co-factor. **B)** AAM but with mean developmental gene expression as co-factor. **C)** Interaction model but with mean developmental gene expression as co-factor. Cyan and red show eQTLs according to the IMM and IMI respectively. Counts are in terms of number of transcripts mapping to the location.


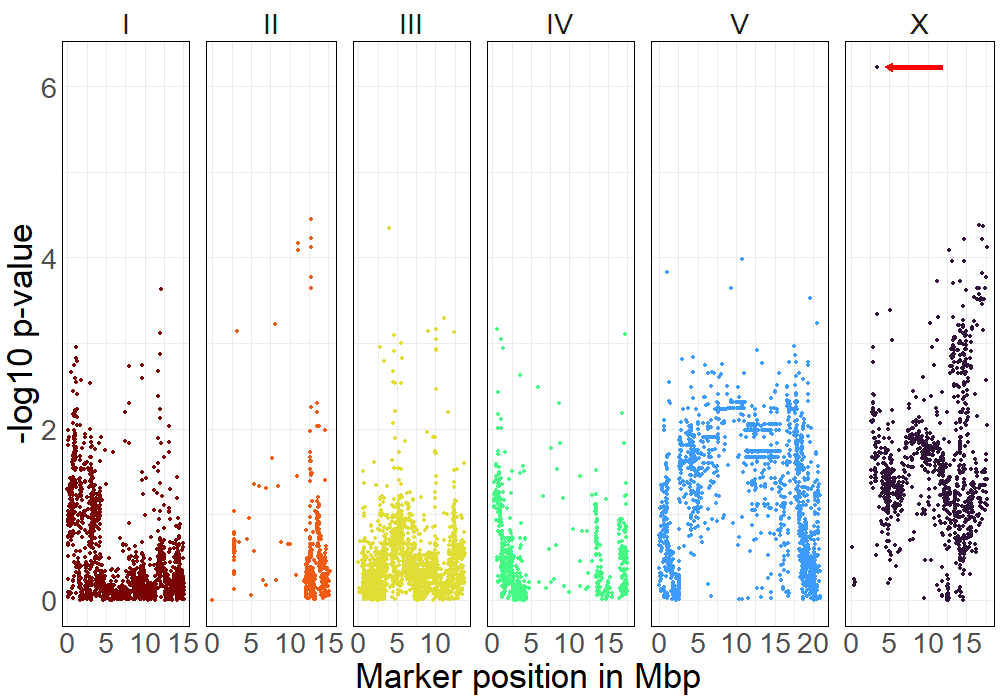


**Figure S9: Hotspot locus is most predictive of PC1.** Marker p-values obtained with a linear model of PC1 ~ marker genotype. The red arrow denotes an outlier corresponding to the marker at 3.404162 Mb on chromosome X. This is also the position of the largest hotspot according to the SMM.


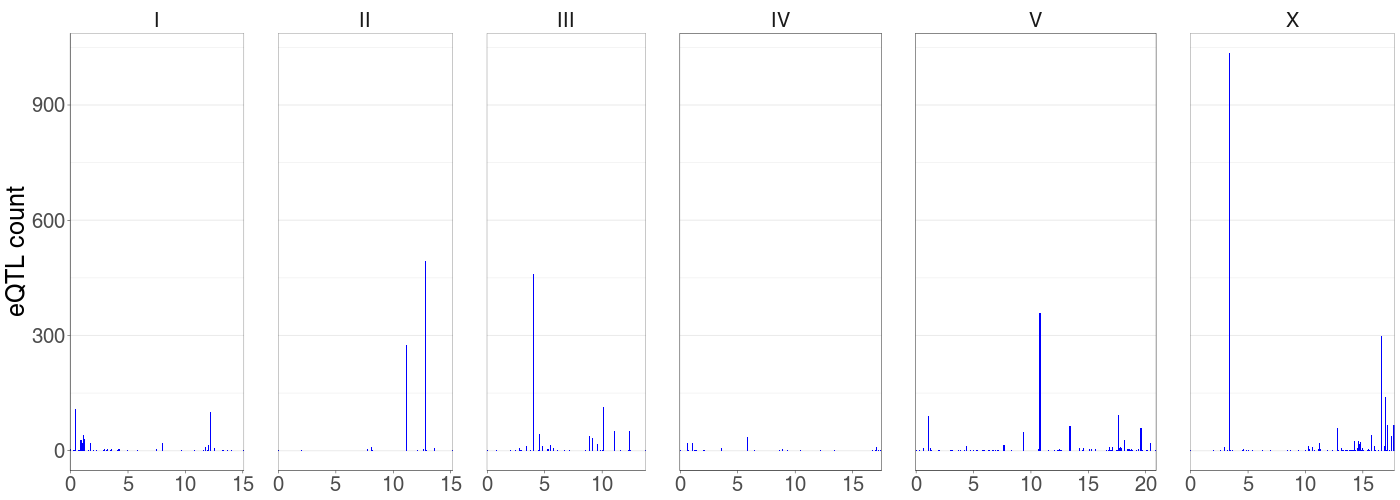


**Figure S10: Distribution of SMM-only eQTLs over the genome.** X-axis shows the position on the genome. Y-axis shows the number of eQTLs that map to this position. Counts are in terms of transcripts mapping to this location.


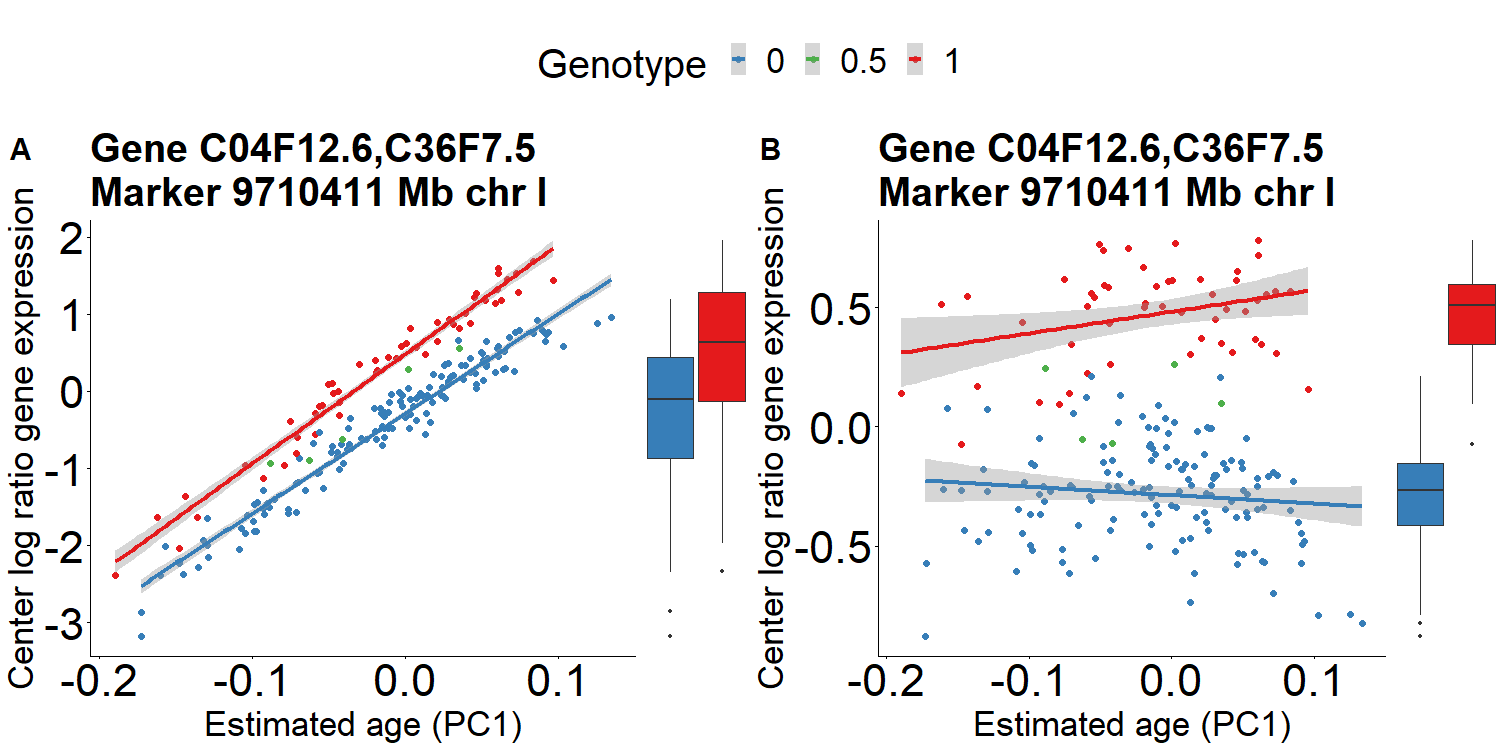


**Figure S11: Gene expression before and after removing developmental variation for an example eQTL.** Color corresponds to genotype at eQTL position. We remove developmental variation by calculating the coefficient of the developmental age variable in the AAM. We then subtract this coefficient multiplied with the projection on PC1 from the expression of each of the mpRILs. Lines are the best fit of IM model (grey area is 95% CI). The boxplots show that the allelic effect on the magnitude of gene expression appears lower **A)** before compared to **B)** after removing developmental variation.


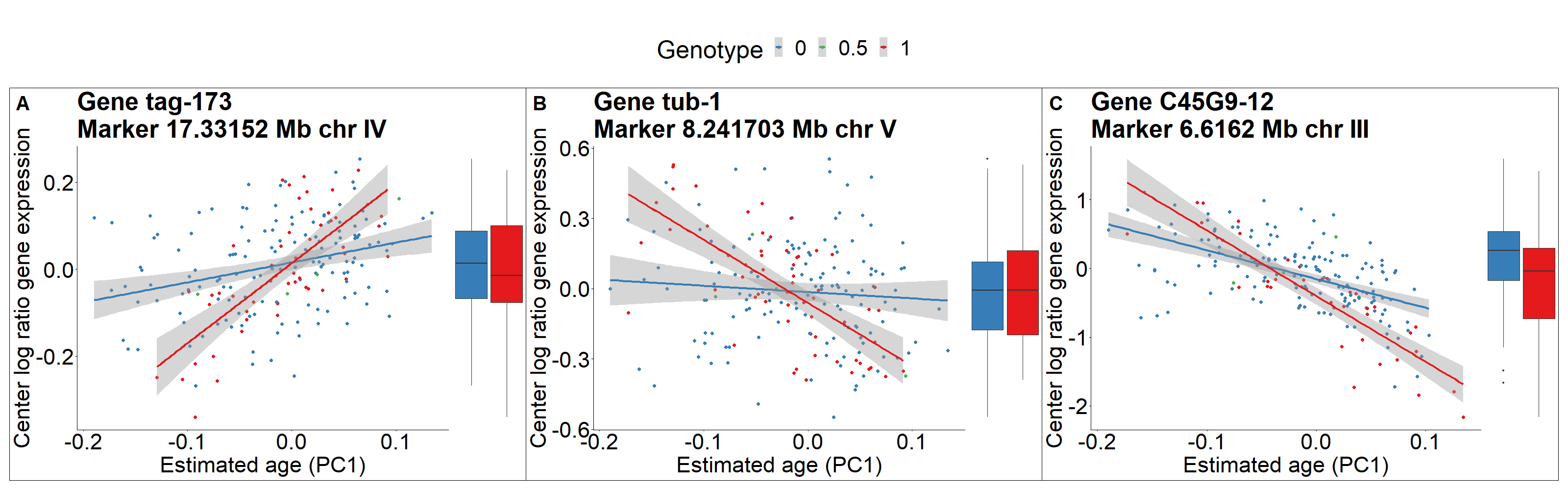
**Figure S12: Examples of eQTLs with an interaction that is significant at 0.1 FDR but not at 0.05 FDR.** Color corresponds to genotype. Lines show the best fit of IM model to the data. Shaded areas show the 95% confidence interval. Boxplots show magnitude of gene expression without developmental context.


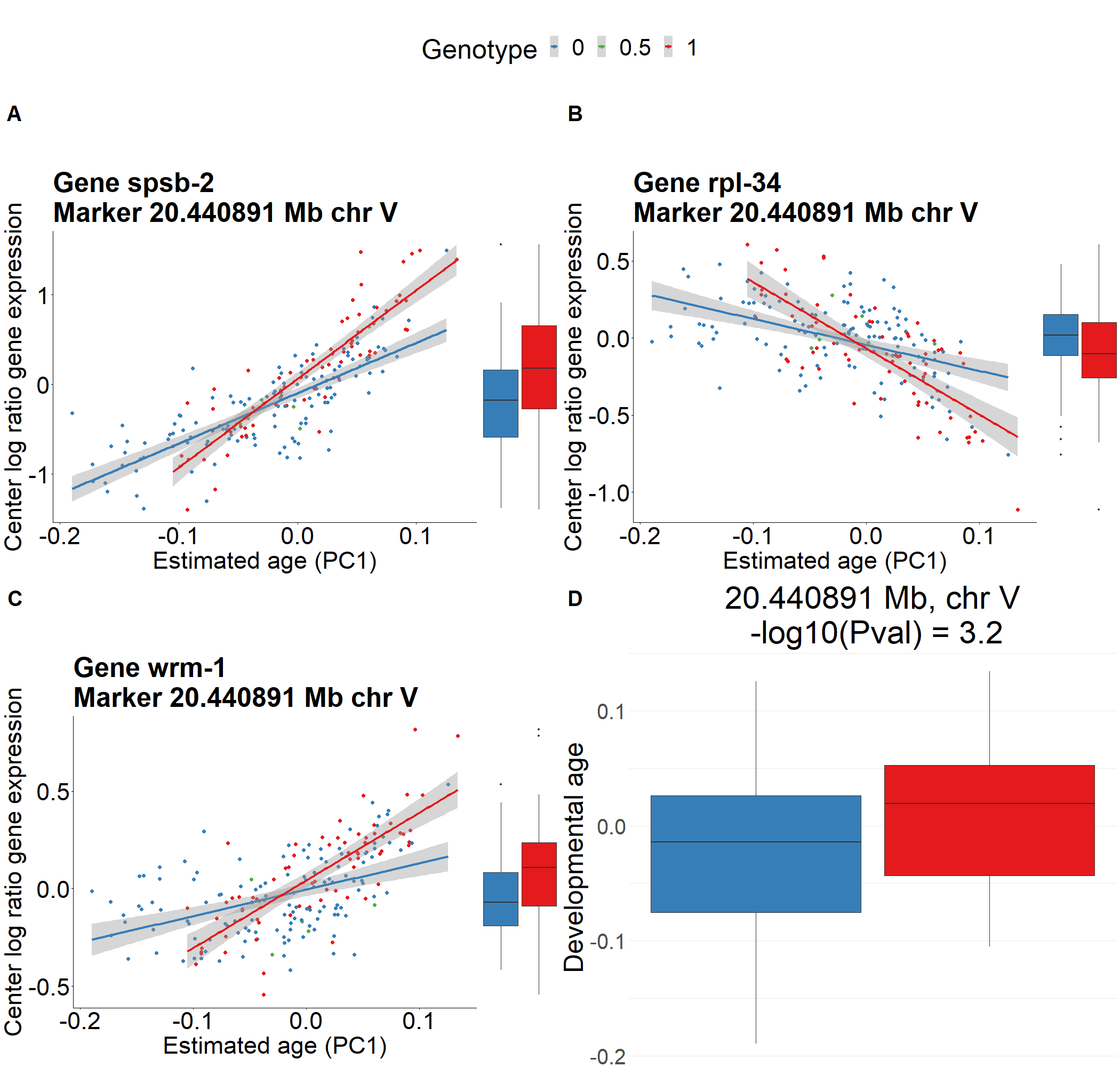


**Figure S13: Interaction hotspot on chromosome V affects the rate at which transcript levels change.** Hotspot location is at 20.440891 Mb. **A-C)** Expression of three example transcripts with a significant interaction eQTL (FDR = 0.05) that maps to the hotspot position. **D)** Boxplot of the developmental age for the alleles at the hotspot marker. The weight of the distribution is skewed towards lower developmental ages for allele 0. P-value is obtained using a linear model of PC1~genotype at hotspot position.


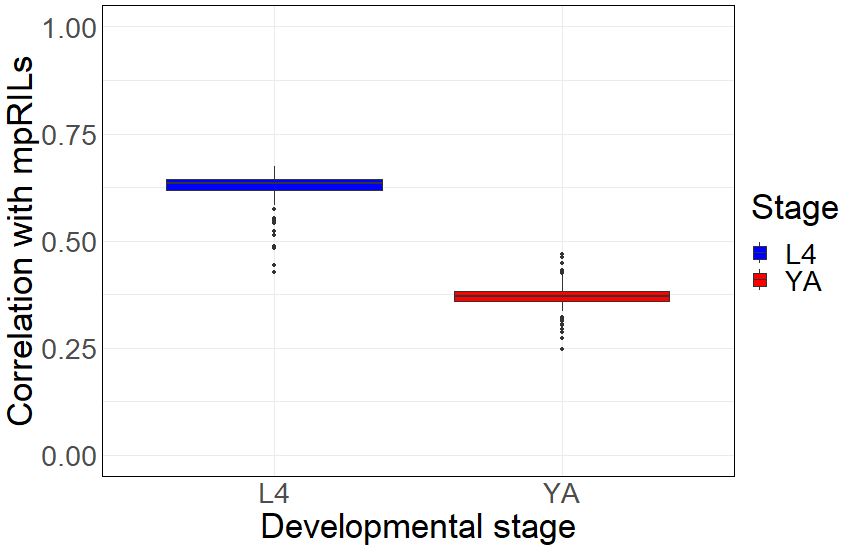


**Figure S14: Correlation transcriptomes mpRILs with L4 and young adult reference transcriptomes.** Boxplot of the mean correlation of the mpRILs with two L4 reference genomes (L4 and L4b from Boeck *et al.*, 2016) (Blue), and the mean correlation with two young adult reference genomes (YA and N2Yad-1 from Boeck *et al.*, 2016) (Red). For each mpRIL the transcriptome shows a stronger mean correlation with the L4 reference transcriptome compared to the correlation with the L3 reference transcriptome.


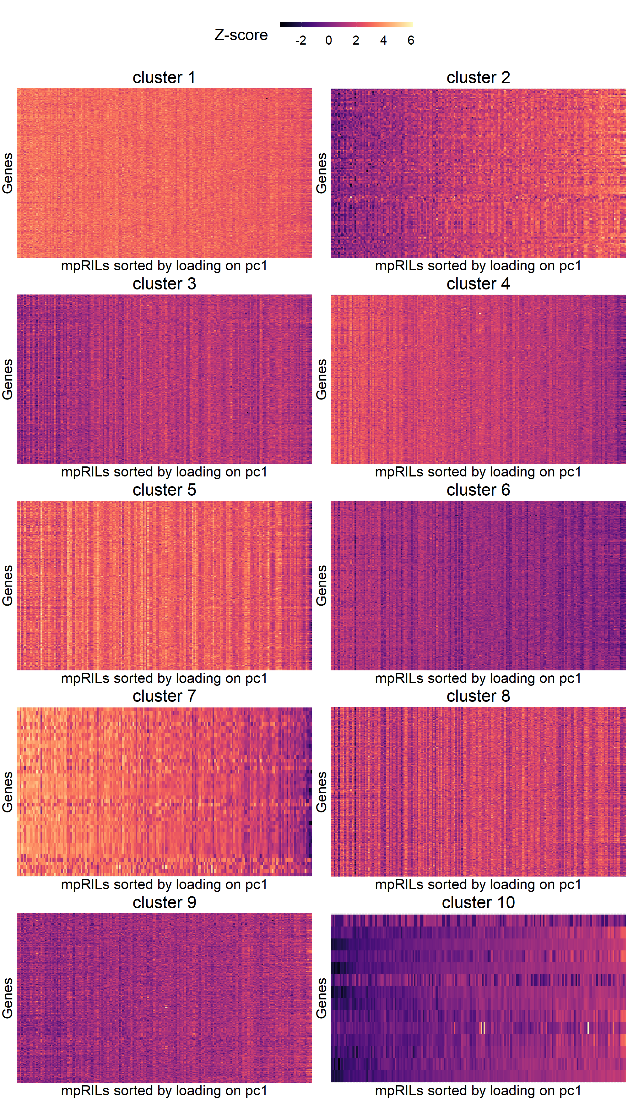

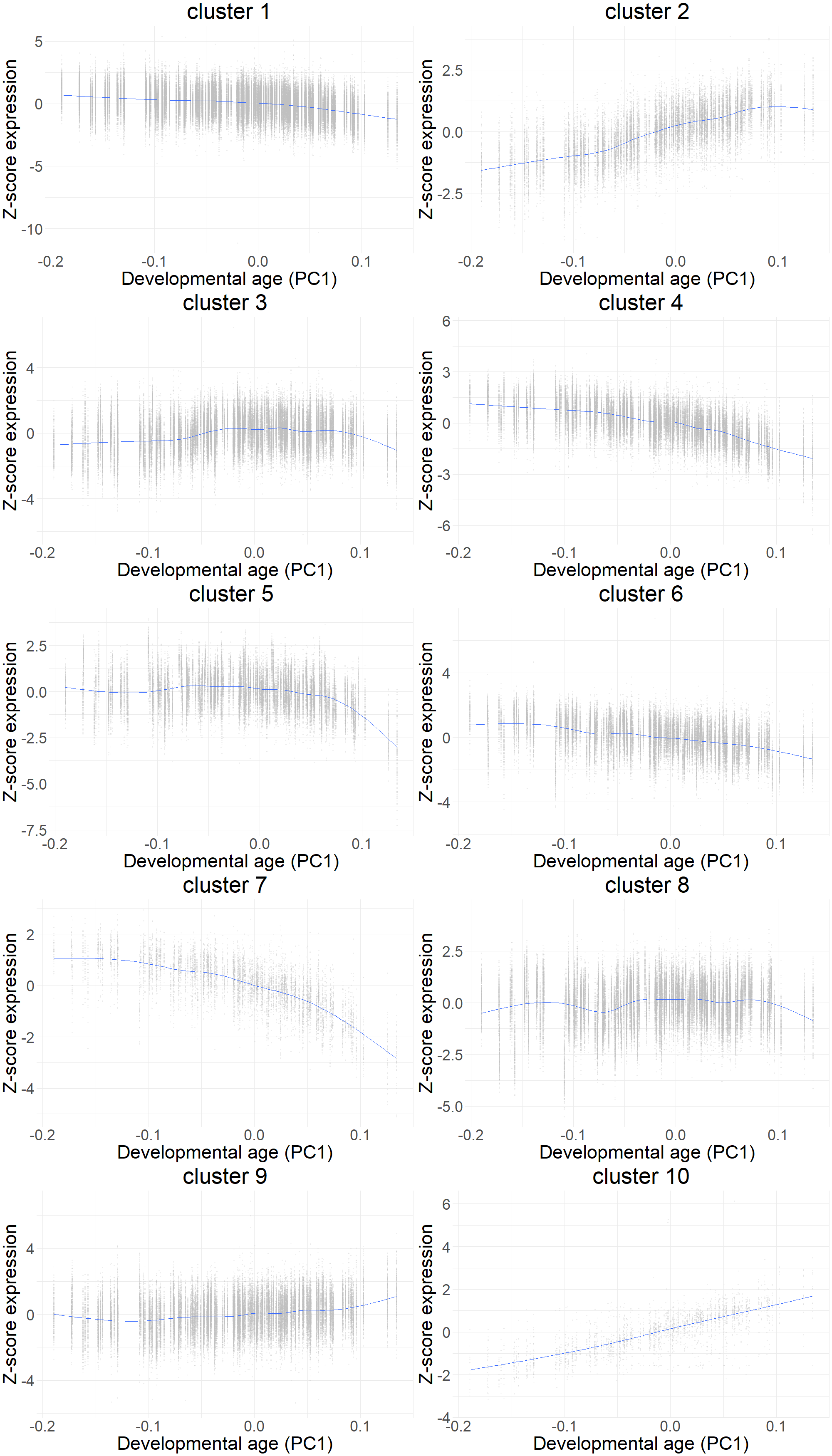


**Figure S15:** Heatmaps and line plots of Z-scores of center log ratio of gene expression for clusters of oscillatory genes(Hendriks et al. 2014). Clusters were determined using k-means clustering (n = 10) on a gene expression matrix of oscillatory genes. In total 2346 oscillatory genes with significant expression in our data were used in the clustering. The number of genes per cluster from cluster 1 to cluster 10 were 562, 124 ,351, 266, 191, 215, 46, 304, 273 and 14.


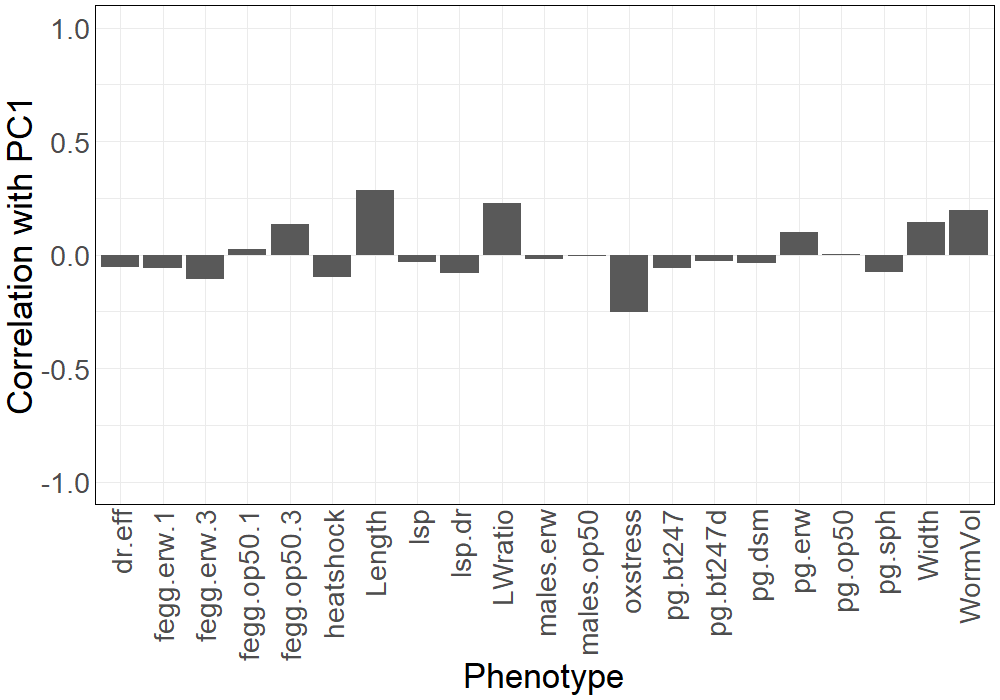


**Figure S16: Correlation between phenotypes scored in** **Snoek *et al.*, 2019 and PC1.** From the method section of Snoek *et al.*, 2019: “Dr.eff is the difference in average life span between NGM and DR medium in days. Fegg.erw.1 is the time in hours until the first egg (1–10) for populations grown on Erwinia. Fegg.erw.3 is the time in hours until the first egg (> 100) for populations grown on Erwinia. Fegg.op50.1 is the time in hours until the first egg (1–10) for populations grown on OP50. Fegg.op50.3 is the time in hours until the first egg (> 100) for populations grown on OP50. Heat shock is the average number of dead animals per 50. Length is in nanometers. Lsp is the average lifespan on NGM in days. lsp.dr is the average lifespan on DR medium in days. LWratio is the length in nanometers divided by the width in nanometers. Oxidative stress indicates activity in terms of movement after addition of hydrogen peroxide. Males.erw and males.op50 is the occurrence of males on plates (0 = none , 0.5 = 1 plate, 1 = 2 plates). Pg.bt247, pgbt247d, pg.dsm, pg.erw, pg.op50 and pg.sph shows population growth (worms per 5 μl of culture) on a pathogenic Bacillus thuringiensis strain NRRL B-18247 on two concentrations of 1:300 and 1:600, a non-pathogenic Bacillus thuringiensis strain DSM-350E, Erwinia rhapontici (isolated from Orsay, France), Escherichia coli OP50 and Sphingobacterium sp. (isolated from Orsay, France). Width is in nanometers. Wormvolume is volume in nanoliters”.
