# Supplementary material for: The effect of developmental variation on expression QTLs in a multi parental Caenorhabditis elegans population
Source: G3 (Bethesda). 2023 Nov 28;14(2):jkad273. doi: 10.1093/g3journal/jkad273 (PMC10849341; doi:10.1093/g3journal/jkad273)
Supplement: jkad273_Supplementary_Data [file jkad273_supplementary_data.zip › Text_S1_G3-2023-404548.docx]

**Text S1: Effect of developmental variation on the narrow sense heritability of gene expression**

*Methods*

We calculated narrow sense heritability using the marker_h2() function from the heritability package. This function requires a kinship matrix as input, which we calculated using the A.mat() function from the rrBLUP package. We calculated the heritability of gene expression both with and without the loadings on PC1 as a covariate.

*Results*

We wondered to what extent the developmental speed of the mpRILs was heritable. To investigate we quantified the narrow sense heritability (h_2_) of PC1, our proxy for the developmental age. Our analysis indicates an h_2_ of ~0.23. The h_2_ of ~0.23 shows that the developmental speed is partially heritable through additive genetic effects. It follows that the h_2_ of gene expression of transcripts involved in or affected by the developmental process can be decomposed into a component that comes from the additive heritability of the developmental speed and the remaining h_2_ that comes from other sources of genetic variation. We wanted to investigate to what extent gene expression differences are inherited through the generic process of development. To this end, we performed a heritability analysis on the expression level of all transcripts, both with and without PC1 as a covariate. We expected that in most cases the heritability of gene expression would be lower when the developmental age was added as a co-factor, because one potential component of h_2_ is now removed. However, we also expected that for some transcripts with AAM-only eQTLs, the “other” component of the h_2_ of gene expression could be partially hidden by variation in developmental age, resulting in underestimation of h_2_ in the absence of the developmental age as a covariate **(Figure 7B, Figure S11**).

Adding developmental age as cofactor had a major impact on the estimated h_2_. For 1210 transcripts the h_2_ increased by more than 0.05 when PC1 was included as a covariate. Conversely, for 4410 transcripts the h_2_ decreased by more than 0.05 when PC1 was included (**Table T1.1**)**.** We investigated if there was an association between the type of eQTLs affecting expression and the difference in h_2_ with and without PC1 (**Figure T1.1**). Transcripts with no eQTLs tend to have low h_2_ with or without PC1 as a covariate, while transcripts with only eQTLs detected by both models tend to have similar h_2_, regardless of correcting for PC1. Transcripts with a larger than 0.2 difference in h_2_ when including PC1 as a covariate almost exclusively (1164 out of 1180) have either an AAM-only eQTL, SMM-only eQTL or both. This demonstrates that, for transcripts with SMM-only or AAM-only eQTLs, the h_2_ of transcript levels strongly depends on variation in developmental age. The direction of the change in h_2_ also differs depending on the type of eQTL. Out of 498 transcripts with an AAM-only eQTL but not a SMM-only eQTL, 234 transcripts have an increase > 0.05 in h_2_ when including PC1, compared to 34 transcripts with a decrease > 0.05 (**Table T1.1**). For transcripts with an SMM-only eQTL but not an AAM-only eQTL the direction is reversed, with 266 transcripts with an increase > 0.05 in h_2_ with PC1 as covariate and 3092 transcripts with a decrease > 0.05.


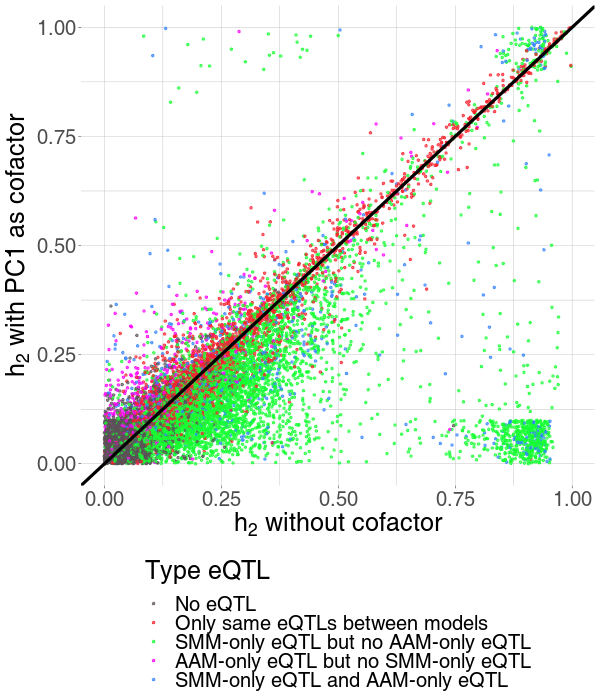


**Figure T1.1: Heritability of gene expression with and without PC1 as co-factor depends on the type of eQTLs.** The x-axis shows the heritability calculated without PC1 as covariate. The y-axis shows the heritability calculated with PC1 as covariate. Each dot represents one of the 12029 transcripts with expression in our dataset. Black line has slope 1 and passes through the origin. Transcripts are colored by whether they have no eQTLs according to the SMM and AAM (orange), the same eQTLs between both models (green), a SMM-only eQTL but not an AAM-only eQTL (light blue), an AAM-only eQTL but not a SMM-only eQTL (brown) or a SMM-only eQTL as well as an AAM-only eQTL (purple).

**Table T1.1: Differences in heritability with and without adding PC1 as a cofactor for subsets of transcripts.** Percentages are in terms of total number of transcripts in the subset (shown in left column).

|  | Total transcripts | Number of genes with 0.05 increase in h_2_ when adding PC1 as covariate | Number of genes with 0.05 decrease in h_2_ when adding PC1 as covariate |
| --- | --- | --- | --- |
| All transcripts | 12029 | 1210  10.1% | 4410  36.7% |
| Transcripts with no eQTL | 2842 | 347  12.2% | 319  11.2% |
| Transcripts with same eQTL between SMM and AAM | 2589 | 244  9.4% | 296  10.4% |
| Transcripts with AAM-only eQTL but not a SMM-only eQTL | 498 | 234  47.0% | 34  6.8% |
| Transcripts with SMM-only eQTL but not an AAM-only eQTL | 5451 | 266  4.9% | 3092  56.7% |
| Transcripts with a SMM-only as well as an AAM-only eQTL | 649 | 119  18.3% | 330  50.8% |

Since eQTLs can be indicators of genomic and therefore heritable regulation of gene expression, the h_2_ of gene expression is expected to increase with the number of eQTLs. This expectation clearly holds in our data if the h_2_ is not corrected for the developmental age (**Figure T1.3A**). However, including PC1 as a cofactor in the heritability calculation results in lower heritability for many transcripts (**Figure T1.1**). Such transcripts tend to have many SMM-only eQTLs (**Figure T1.1, compare Figure T1.2A and T1.2B**), which result from developmental variation in the population (**Figure 7A**). Therefore, we hypothesized that the positive relationship between the number of eQTLs and the h_2_ should be reduced if the h_2_ is corrected for the developmental age. This is indeed what we observe for the SMM (**Figure T1.3B**). However, because eQTLs detected by the AAM do not depend on developmental variation (**Figure 7B**), the positive relationship between the number of eQTLs when using the AAM and the h_2_ of gene expression is robust to developmental correction of the h_2_ (**Figure T1.3C, D**).


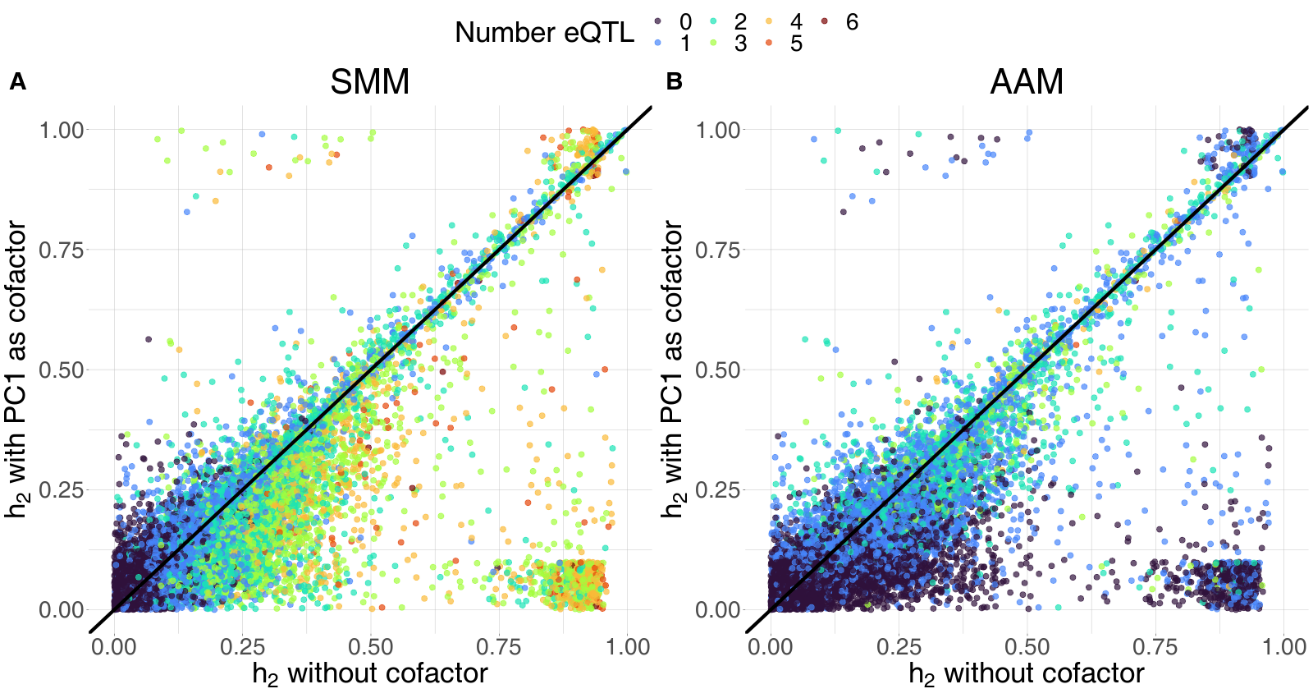


**Figure T1.2: Relationship between heritability of gene expression with and without PC1 as co-factor and the number of eQTLs with the SMM and AAM.** The x-axis shows the heritability calculated without PC1 as covariate. The y-axis shows the heritability calculated with PC1 as covariate. Each dot represents one of the 12029 transcripts with expression in our dataset. Black line has slope 1 and passes through the origin. Transcripts are colored by the number of eQTL affecting their expression. Values range from 0 to 6 because we call a maximum of one eQTL per chromosome. **A)** Number of eQTLs detected with SMM. **B)** Number of eQTLs detected with AAM.


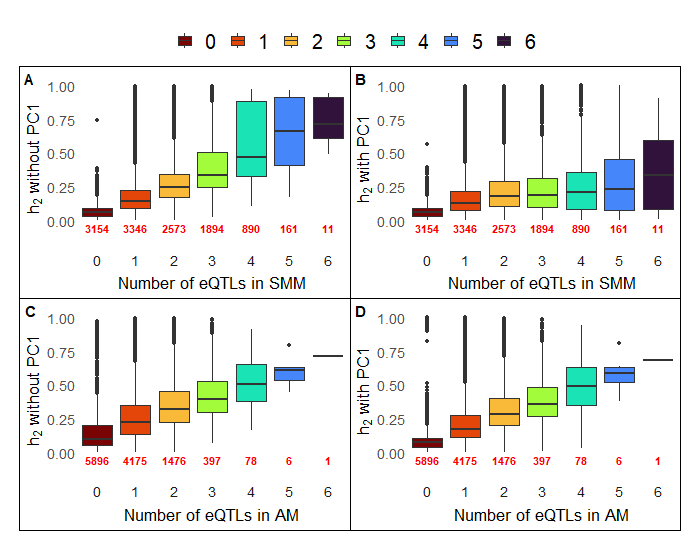


**Figure T1.3: Relationship between the number of eQTLs and the h_2_.** In each subfigure we show the distribution of the h_2_ for transcripts with 0 to 6 eQTLs. Between the subfigures we show how these distributions change if we correct for development in the mapping, in the heritability calculation itself or in both. **A)** Relationship between the number of eQTLs detected by the SMM and the h_2_ of gene expression. **B)** Relationship between the number of eQTLs detected by the SMM and the development corrected h_2_. **C)** Relationship between the number of eQTLs detected by the AAM and the h_2_. **D)** Relationship between the number of eQTLs detected by the AAM and the development corrected h_2_.

*Discussion*

The relatively low h_2_ of PC1 (~0.23) was surprising to us, given that the parental duplicates have similar projections on PC1 **(Figure S5)**, suggesting that the genetic background of the mpRILs is an important cause of developmental age variation. A potential explanation for these contrasting results could be that epistatic interactions are important determinants of the developmental age, whereas the narrow sense heritability considers only additive genetic effects. We calculated the narrow sense heritability (h_2_) of gene expression both with and without PC1 as cofactor. Comparing the standard h_2_ with the development corrected h_2_ could provide an indication of whether a gene’s expression is in part heritable through the genetical component of developmental age differences, or mostly independent from developmental age. In the first case one would expect the development-corrected h_2_ to decrease compared to the standard h_2_. On the other hand, if the heritability of gene expression is independent from the developmental process, one would expect the development-corrected heritability to be similar or higher compared to the standard h_2_. While the developmental age is itself heritable and can thus have a real effect on the heritability of expression, it can also confound heritability analysis. This is evidenced by the subset of transcripts with a much larger development corrected h_2_ compared to standard h_2_ (**Figures T1.1, T1.2**). Possibly, this confounding effect can be explained by the relatively low h_2_ of PC1. A large part of the variation in developmental age could be due to stochastic factors such as small fluctuations in temperature within the experimental setup(Mata-Cabana et al. 2022) or non-additive genetic effects. This part of developmental variation could obscure the effect of the genomic background on the h_2_ of gene expression for some transcripts, resulting in more accurate heritability estimates when controlling for development.
