# Supplementary material for: The effect of developmental variation on expression QTLs in a multi parental Caenorhabditis elegans population
Source: G3 (Bethesda). 2023 Nov 28;14(2):jkad273. doi: 10.1093/g3journal/jkad273 (PMC10849341; doi:10.1093/g3journal/jkad273)
Supplement: jkad273_Supplementary_Data [file jkad273_supplementary_data.zip › Text_S2_G3-2023-404548.docx]

**Text S2: Allele dependent non-linear gene expression dynamics**

*Methods*

To detect allele dependent non-linear gene expression patterns, we performed eQTL mapping with natural splines. We used the ns() function from the splines package to model the dynamics over the developmental age. We used three knots, chosen such that each interval characterized by the knots contains one fourth of the mpRILs. We obtained p-values by performing a two model anova using the anova() function (stats package, base R). One of the models included in the anova contained an interaction between the marker and a natural spline of the developmental age while the other contained only an additive contribution of the natural spline. We used a cutoff of –log10(P) = 7.53 resulting in an FDR of 0.2, as using an FDR of 0.1 (-log10(P) = 12.1) only resulted in 25 significant interactions, while it was impossible to threshold at an FDR of 0.05.

*Results*

Gene expression dynamics can be more complex than simple linear trajectories. To investigate the prevalence of allele dependent non-linear relationships between gene expression and PC1 we performed eQTL mapping using a natural spline of the developmental age(Francesconi and Lehner 2014). To search for non-linear interactions between the developmental age and the genotype we used a cutoff of –log10(P) > 7.53 (FDR = 0.2) for the interaction term of the model. More stringent cutoffs detect very few interactions. The 0.2 FDR threshold indicated the presence of 113 potential non-linear gene expression patterns **(Figure T2.1**). Furthermore, we found multiple qualitatively similar expression patterns in transcripts associated with the same eQTL **(Figure T2.2**), demonstrating that complex dynamics can be heritable.

To quantify the effect of eQTL detection with the natural spline model we divided the 113 eQTLs into categories **(Figure T2.3**). The first category (55 eQTLs) contains those eQTLs which clearly result from highly uneven genotype distributions or just a few outliers. Uneven distributions or outliers can lead to overfitting because of the flexibility of the natural spline model. Furthermore, due to the multi-parental origin of the mpRILs, uneven allelic distributions are prevalent in our data. The second category (25 eQTLs) corresponds to interactions that were approximately linear. Such gene expression patterns are also adequately described by a linear interaction model. A third category (11 eQTLs) contains eQTLs with a non-linear but approximately monotonic gene expression pattern. Finally, the fourth category (22 eQTLs) consists of non-monotonic gene expression patterns. Especially eQTLs in this latter category demonstrate the added value of natural splines, as complex patterns can average out to similar mean expression values (**Figure A2.1C**).
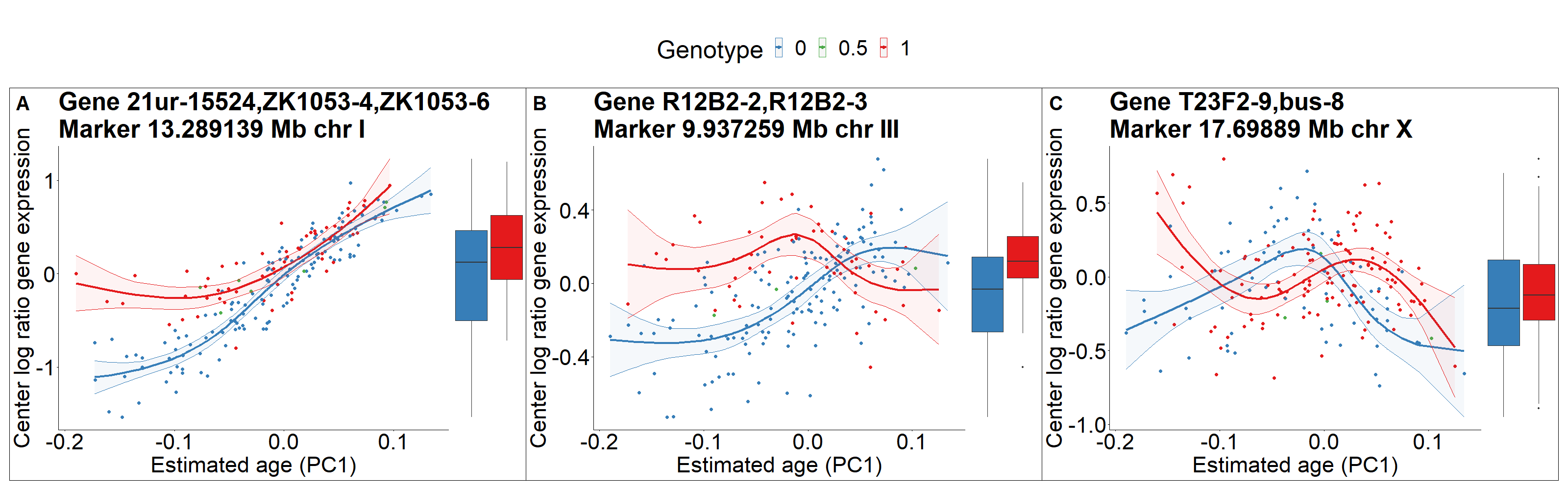


**Figure T2.1: Example non-linear gene expression patterns detected using natural splines.** Colors correspond to genotype at eQTL position. Lines are best fit of natural spline model (shaded area is 95% CI) and show the dynamics of gene expression. Boxplots show magnitude of gene expression without temporal component. **A)** eQTL that causes allele-dependent monotonic expression dynamics at the lower end of the developmental age range of the mpRILs. **B)** eQTL for which one genotype shows a monotonic increase over the developmental age while the other shows non-monotonic concave dynamics. **C)** eQTL where both genotypes show different complex, non-monotonic dynamics.


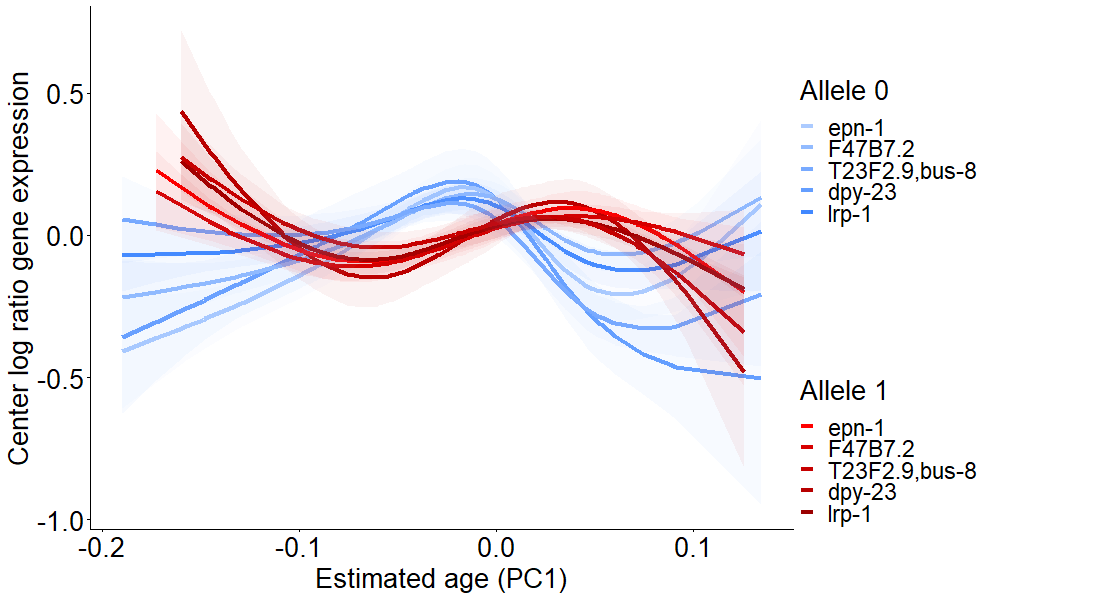


**Figure T2.2: Example of marker causing consistent dynamics for multiple transcripts.** Fit of the natural spline model to the center log ratio of gene expression of transcripts with a –log10(interaction p-value) >7.5 in the spline models with the adjacent markers at 17.519998 Mb chr X (*F47B7.2*; *lrp-1*) or 17.69889 Mb chr X (*epn-1*; *T23F2.9*, *bus−8*; *dpy-23*).


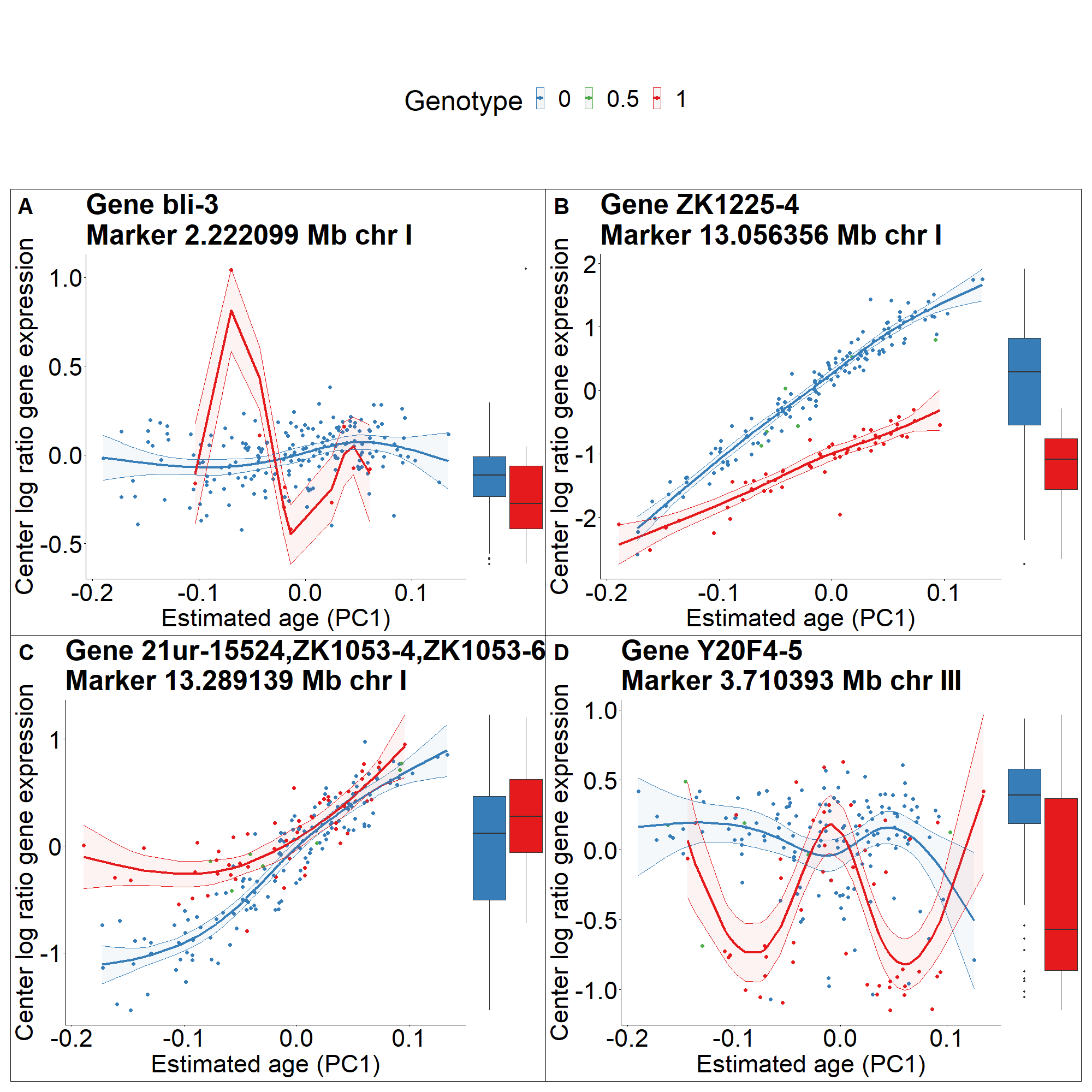


**Figure T2.3:** Four categories of eQTLs detected using the interaction term of the natural spline model at 0.2 FDR. Category was scored by eye from a total of 113 eQTLs. **A)** False positive due to uneven genotype distribution/outlier. **B)** Approximately linear interaction. **C)** Monotonic non-linear interaction. **D)** Non-monotonic interaction. We excluded from the scoring two significant markers for which almost all mpRILs were heterozygous.
